# Supplementary material for: LATE–a novel sensitive cell-based assay for the study of CRISPR/Cas9-related long-term adverse treatment effects
Source: Mol Ther Methods Clin Dev. 2021 Jul 29;22:249–62. doi: 10.1016/j.omtm.2021.07.004 (PMC8399046; doi:10.1016/j.omtm.2021.07.004)
Supplement: Document 1. Table S1 and Figures S1–S7 [file mmc1.pdf]

OMTM, Volume *22*

Supplemental information

LATE–a novel sensitive cell-based assay for the study of CRISPR/Cas9-related long-term adverse treatment effects

Dawid Głów, Simon Meyer, Irene García Roldán, Lara Marie Akingunsade, Kristoffer Riecken, and Boris Fehse

Table S1. Oligonucleotides and oligonucleotide probes used in the study.

R NAME	SEQUENCE	REMARKS
Fspcas9K855A	GCGGTGCTGACCAGAAG	PCR primers for introduction K855A substitution into Cas9 coding sequence
Rspcas9K855A	GTTGTCGATGGAGTCGTCC	
Fspcas9K810A	GCGCTGTACCTGTACTACCTGCAG	PCR primers for introduction K810A substitution into Cas9 coding sequence
Rspcas9K810A	CTCGTTCTGCAGCTGGGTG	
Fspcas9K1003A	GCGCTGGAAAGCGAGTTC	PCR primers for introduction K1003A substitution into Cas9 coding sequence
Rspcas9K1003A	AGGGTACTTTTTGATCAGGGCG	
Fspcas9R1060A	GCGCCTCTGATCGAGACAAACG	PCR primers for introduction R1060A substitution into Cas9 coding sequence
Rspcas9R1060A	CTTCCGGATCTCGCCGTTGG	
Fcyp1a1-2	ACCGATTGGGCACATGCTGACCC	Contain CYP1A1 targeting protospacer sequence
Rcyp1a1-2	AACGGGTCAGCATGTGCCCAATC	
FcypBOTH	ACCGACCCGCACCTGGCACTGTCA	Contain CYP1A1 and CYP1A2 targeting protospacer sequence
RcypBOTH	AACTGACAGTGCCAGGTGCGGGTC	
fCYP1A1	ACCGACAGAAGATGACAGAGGCC	Contain CYP1A1 targeting protospacer sequence
rCYP1A1	AACGGCCTCTGTCATCTTCTGTC	
fSOX11	ACCGTGCAGGAGCTGCTGGTGCGG	Contain SOX1 targeting protospacer sequence
rSOX11	AACCCGCACCAGCAGCTCCTGCAC	
fGFOD1	ACCGGAGCAGGAGCTGCTGGTGC	Contain GFOD1 targeting protospacer sequence
rGFOD1	AACGCACCAGCAGCTCCTGCTCC	
fCYP1A2	ACCGGCAGAAGATGGCAGAGGCC	Contain CYP1A2 targeting protospacer sequence
rCYP1A2	AACGGCCTCTGCCATCTTCTGCC	
fRFX1	ACCGAAGCCGGCGCTGCCCTGGT	Contain RFX1 targeting protospacer sequence
rRFX1	AACACCAGGGCAGCGCCGGCTTC	
fOBSL1	ACCGCAGCCGTAGGTGCCCTGGT	Contain OBSL1 targeting protospacer sequence
rOBSL1	AACACCAGGGCACCTACGGCTGC	
fTP53	ACCGACGGAAACCGTAGCTGCCC	Contain TP53 targeting protospacer sequence
rTP53	AACGGGCAGCTACGGTTTCCGTC	
fARID1A	ACCGCCGGACCTGAAGAACTCGAA	Contain ARID1A targeting protospacer sequence
rARID1A	AACTTCGAGTTCTTCAGGTCCGGCGGT	

fSMARCB2	ACCGACGGCGAGTTCTACATGAT	Contain SMARCB2 targeting protospacer sequence		
rSMARCB2	AACATCATGTAGAACTCGCCGTCGGT			
fPTEN	ACCGCTAACGATCTCTTTGATGA	Contain PTEN targeting protospacer sequence		
rPTEN	AACTCATCAAAGAGATCGTTAGC			
fBRCA1	ACCGCTTGTGCTGACTTACCAGAT	Contain BRCA1 targeting protospacer sequence		
rBRCA1	AACATCTGGTAAGTCAGCACAAGC			
fp21	ACCGCGCGACTGTGATGCGCTAA	Contain p21 targeting protospacer sequence		
rp21	AACTTAGCGCATCACAGTCGCGC			
fPLZF2	ACCGTGTGCAAGGCCAACCAGATG	Contain PLZF2 targeting protospacer sequence		
rPLZF2	AACCATCTGGTTGGCCTTGCACAC			
fPLZF2-2MM	ACCGAGAGCAAGGCCAACCAGATG	Contain PLZF2 off-targeting (2MM) protospacer sequence		
rPLZF2-2MM	AACCATCTGGTTGGCCTTGCTCTC			
fPLZF2-3MM	ACCGAGAGGAAGGCCAACCAGATG	Contain PLZF2 off-targeting (3MM) protospacer sequence		
rPLZF2-3MM	AACCATCTGGTTGGCCTTCCTCTC			
		5' modification	3' modification	REMARKS
FddP53	CCTGCACCAGCAGCTCCTAC	-	-	Used for GEF-dPCR TP53 exon-4 mutation
RddP53	CAACTGACCGTGCAAGTCACA	-	-	
HexP53	TGGGCTTCTTGCATTCTGGGACAGC	HEX	BHQ	
FamRFXobsl-p53	CCTACCAGGGCAGCTACGGTTTCCGT	FAM	BHQ	
FamP53-p53	AGCTGCCCTGGTAGGTTTTCTGGGAAG	FAM	BHQ	
FamCYP1-p53	TGGCCCCTGTCATCTTCTGTCCCTTC	FAM	BHQ	
AMPLICON NGS PRIMERS				
FngsTP53ex4	ACACTCTTTCCCTACACGACGCTCTTCCGATCTCCTGCACCAGCAGCTCCTAC			
FngsTP53ex4	GACTGGAGTTCAGACGTGTGCTCTTCCGATCTCAACTGACCGTGCAAGTCACA			

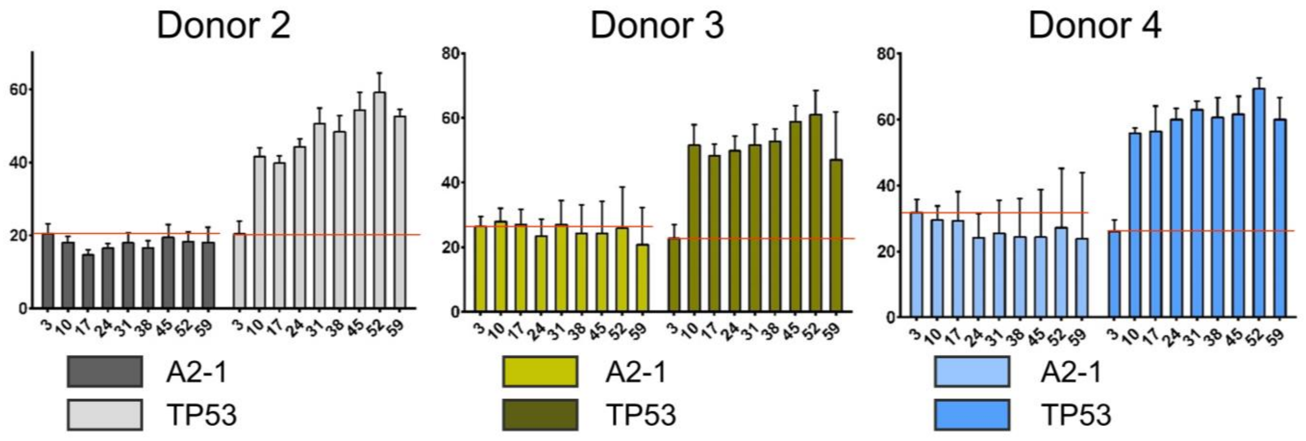

Supplementary Figure S1. LATE-assay readout is not depended on donor specific features. Graph shows Results of the FC analysis of the NUFF from three different donors, transduced with all-in-one lentiviral particles encoding eGFP, wtCas9 and TP53 or A2-1 gRNAs. Red lines mark initial transduction rate. Increase in GFP+ cells can be observed in case of all of the cells transduced with TP53 targeting lentiviruses.

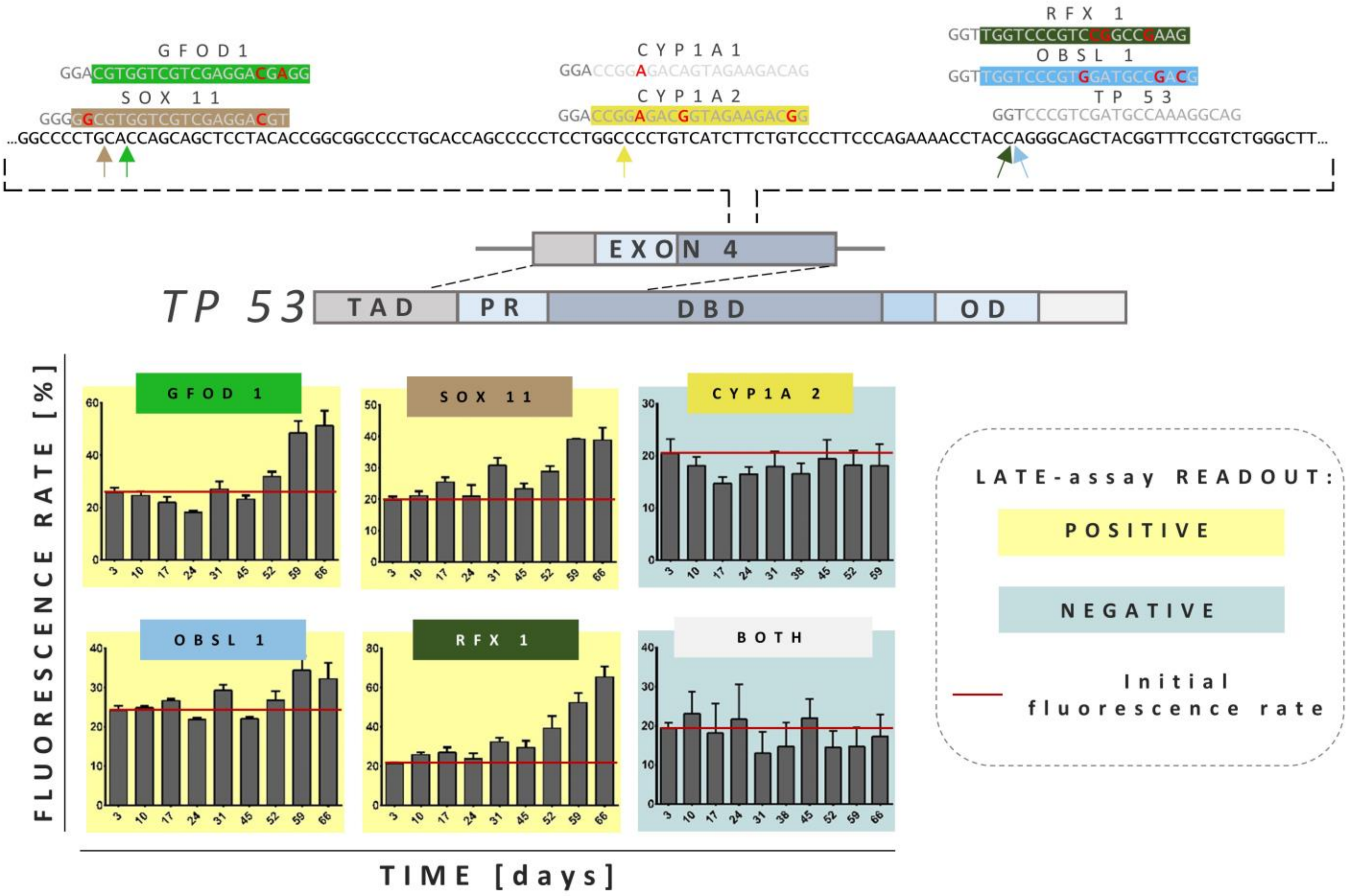

Supplementary Figure S2. LATE-assay is able to detect growth-promoting genome editing events. A) Schematic representation of the TP53 gene and part of the spacer sequences applied to validate LATE-assay (listed in Table 1). Mismatches to the TP53 sequence were marked in red. Cas9 cleavage sites are marked with arrows which colors indicate adequate spacers. B) Comparison of the FC analysis of the NUFF cells transduced with all-in-one lentiviral particles encoding different spacer sequences (OBSL, RFX or A2-1, CYP-BOTH, SOX, GFOD) marked with adequate colors above the graphs. Red lines mark initial transduction rate. Based on the LATE-assay principles, positive and negative LATE results were marked with yellow and blue-grey colors respectively. n=3

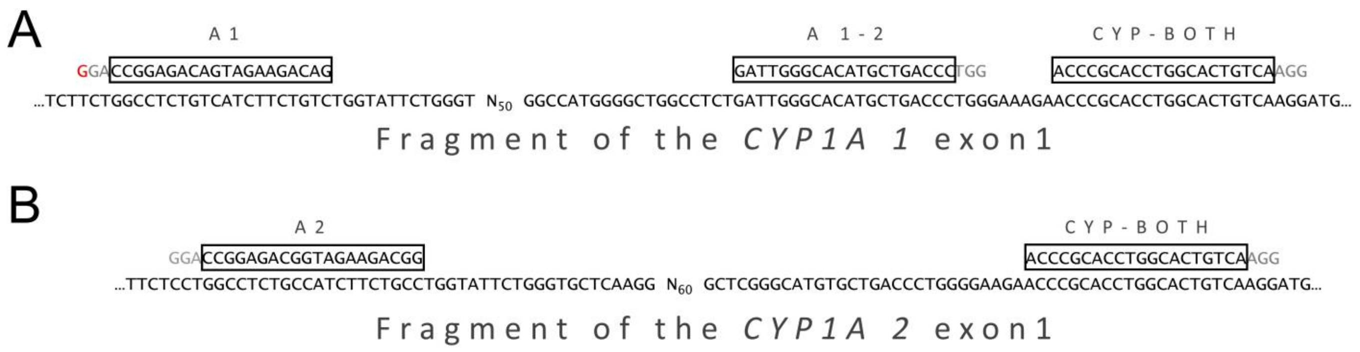

Supplementary Figure S3. Schematic representation of the CYP1A1 and CYP1A2 genes fragments to which spacer sequences were designed.

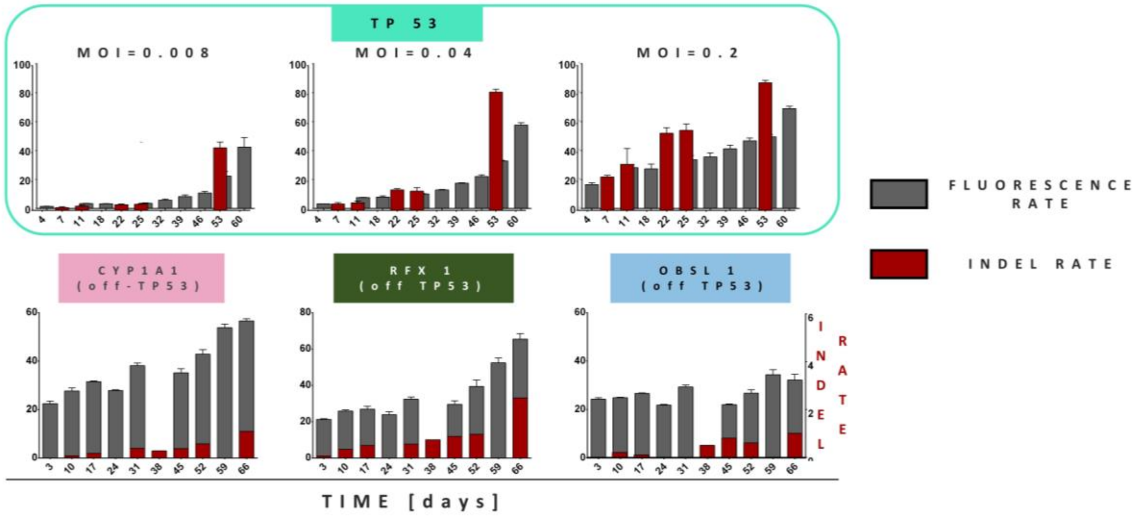

Supplementary Figure S4. Increase in fluorescence rate of the cells transduced with all-in-one lentiviral particles encoding different spacer sequences (TP53, CYP1A1(off TP53), RFX(off TP53) and OBSL(off TP53)) is correlated with indel rate measured by ddPCR. Fluorescence rate is shown in grey and indel rate is shown in red. Only the results OBSL(off TP53) were shown on two different Y axe scales.

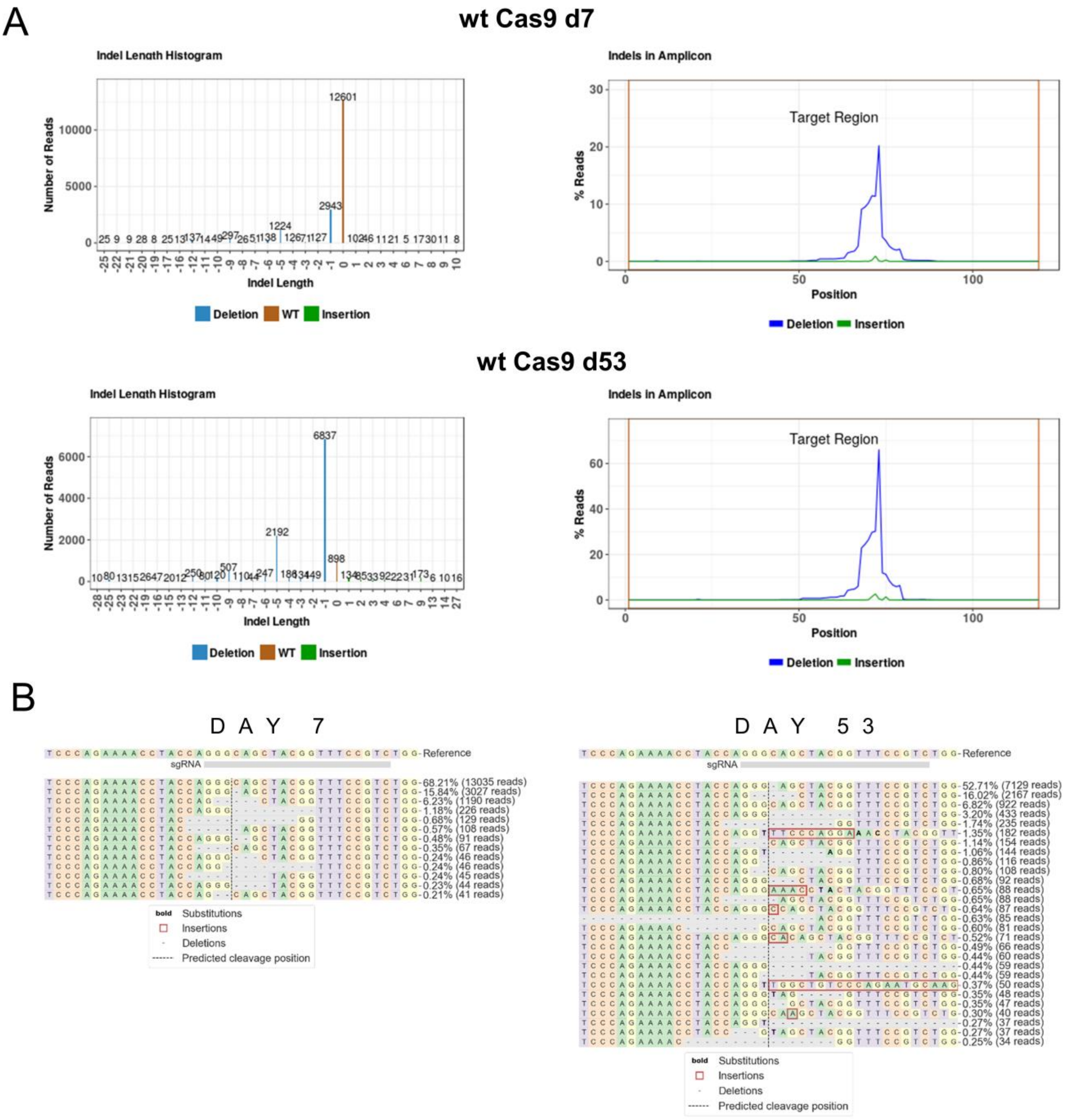

Supplementary Figure S5. NHEJ-mediated Indels within TP53 exon 4 leads to gain of the growth advantage by NUFF cells. **A)** Graphs show Indel length histograms and frequency of the Indels on day 7 and 53 after initial treatment with wt Cas9 and TP53 gRNA. **B)** Graphs obtained with CRISPResso2 shows quality and quantity of Indels found in more than 0.2% of reads day 7 and 53 after initial treatment with wt Cas9 and TP53 gRNA

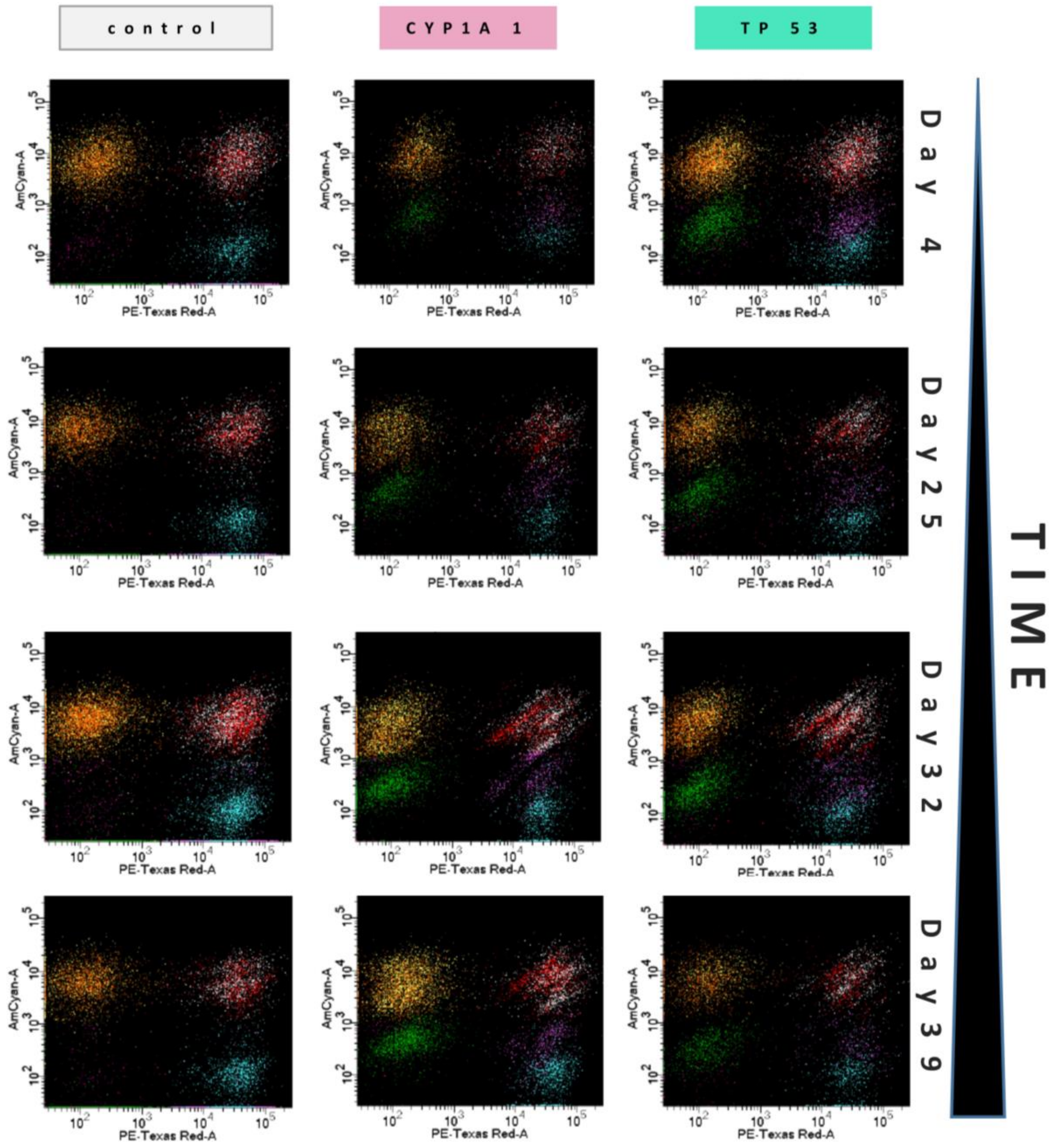

Supplementary Figure S6. Growth advantage gained by the NUFF cells is expressed in clonal growth advantage. Flow cytometry analysis of RGB-marked NUFF cells on week 1, 4, 5, and 6 after initial treatment with wt Cas9 combined with TP53 or A1-1 gRNA. Dominating clones appear on the week 4 in the samples transduced with A1-1 and TP53 gRNAs, however not in the control sample. Clonal domination becomes more pronounced in week 6.

A

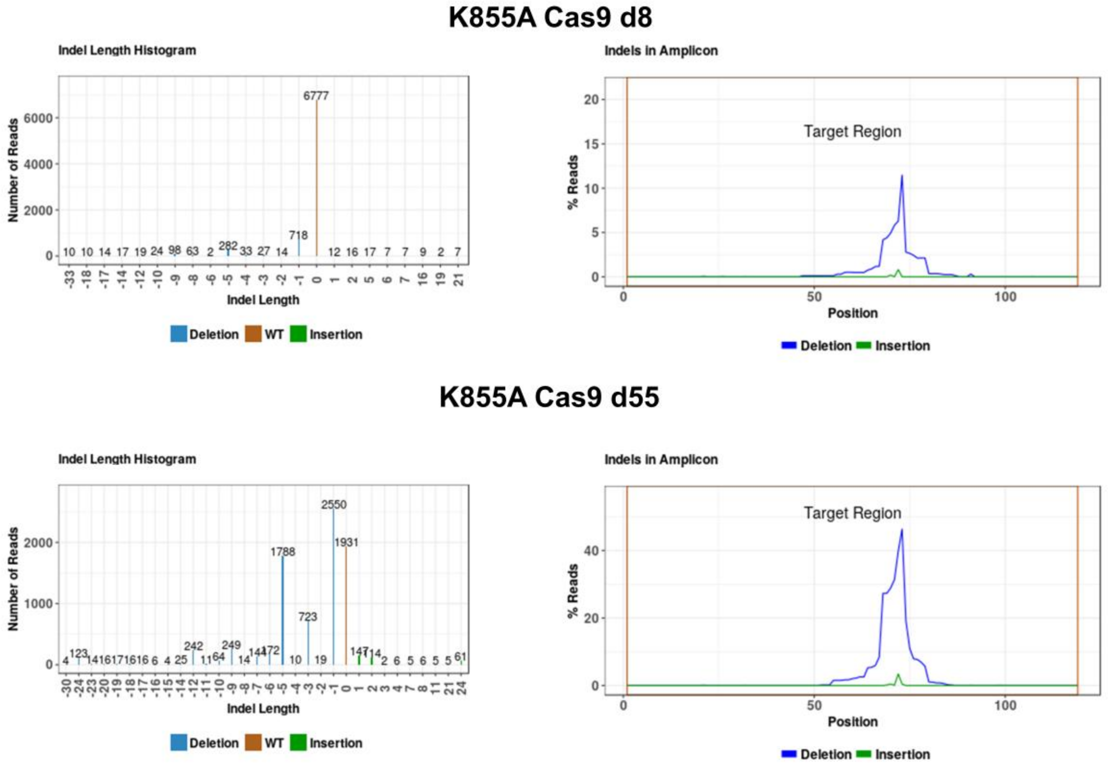

B

	Indel length	Frequency at day 8	Frequency at day 55	Change in %
...GGCCCCTGTCATCTTCTGTCCCTTCCCAGAAAACCTACCAGGGCAGCTACGGTTTCCGTCTGGGCTT...	WT	81.87	21.98	-59.89
...GGCCCCTGTCATCTTCTGTCCCTTCCCAGAAAACCTACCAGGG-AGCTACGGTTTCCGTCTGGGCTT...	-1	8.57	28.18	+19.61
...GGCCCCTGTCATCTTCTGTCCCTTCCCAGAAAACCTACC-----AGCTACGGTTTCCGTCTGGGCTT...	-5	3.01	19.65	+16.64
...GGCCCCTGTCATCTTCTGTCCCTTCCCAGAAAACCTACCAGGG---------TTTCCGTCTGGGCTT...	-9	0.95	2.54	+1.59
...GGCCCCTGTCATCTTCTGTCCCTTCCCAGAAAACCTACCAGGG---CTACGGTTTCCGTCTGGGCTT...	-3	0.32	8.32	+8
...GGCCCCTGTCATCTTCTGTCCCTTCCCAGAAAAC------------CTACGGTTTCCGTCTGGGCTT...	-12	0.23	2.71	+2.41

C

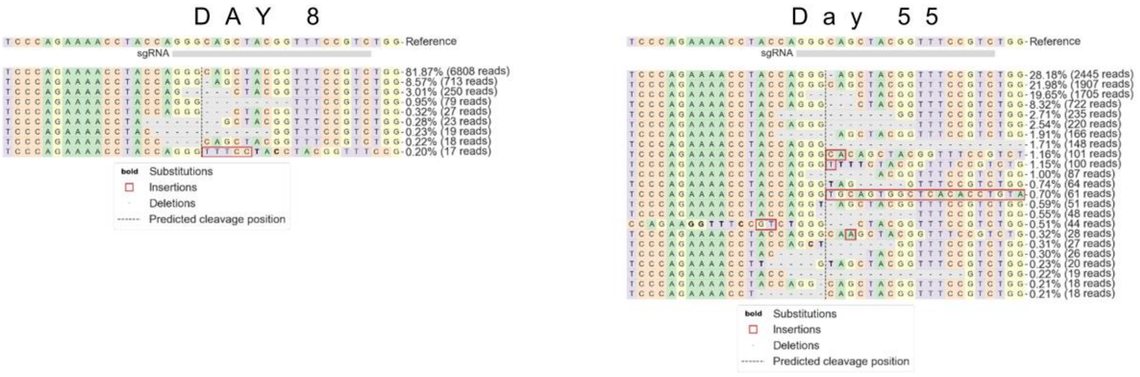

Supplementary Figure S7. Next-generation Cas9 variant's cleavage leads to NHEJ-mediated Indels formation within TP53 exon 4 responsible for the gain of the growth advantage by the NUFF cells. **A)** Graphs show Indel length histograms and frequency of the Indels on day 7 and 53 after initial treatment with Cas9 K855A and TP53 gRNA**. B)** The 5 most frequently found clones on day 8 and 55 are marked with dark gray. Observed change in frequency between day 7 and 53 was marked in blue if positive and brown if negative. **C)** Graphs obtained with CRISPResso2 shows quality and quantity of Indels found in *TP53* in more than 0.2% of NGS reads at day 8 and 55 after initial treatment with Cas9 K855A and TP53 gRNA.
