# Supplementary material for: LATE–a novel sensitive cell-based assay for the study of CRISPR/Cas9-related long-term adverse treatment effects
Source: Mol Ther Methods Clin Dev. 2021 Jul 29;22:249–62. doi: 10.1016/j.omtm.2021.07.004 (PMC8399046; doi:10.1016/j.omtm.2021.07.004)
Supplement: Document 2. Article plus supplemental information [file mmc2.pdf]

Molecular Therapy
Methods & Clinical Development
Original Article

LATE–a novel sensitive cell-based assay for the study of CRISPR/Cas9-related long-term adverse treatment effects

Dawid Głów,[1] Simon Meyer,[1] Irene García Roldán,[1] Lara Marie Akingunsade,[1] Kristoffer Riecken,[1] and Boris Fehse[1]

[1]Research Department Cell and Gene Therapy, Department of Stem Cell Transplantation, University Medical Centre Hamburg-Eppendorf (UKE), Martinistr. 52, Hamburg 20246, Germany

Since the introduction of clustered regularly interspaced short palindromic repeats (CRISPR)/CRISPR-associated protein 9 (Cas9), genome editing has been broadly applied in basic research and applied biotechnology, whereas translation into clinical testing has raised safety concerns. Indeed, although frequencies and locations of off-target events have been widely addressed, little is known about their potential biological consequences in large-scale long-term settings. We have developed a long-term adverse treatment effect (LATE) *in vitro* assay that addresses potential toxicity of designer nucleases by assessing cell transformation events. In small-scale proof-of-principle experiments we reproducibly detected low-frequency (<0.5%) growth-promoting events in primary human newborn foreskin fibroblasts (NUFF cells) resulting from off-target cleavage in the *TP53* gene. Importantly, the LATE assay detected not only off-target effects in *TP53* not predicted by popular online tools but also growth-promoting mutations in other tumor suppressor genes, such as *p21* and *PLZF*. It convincingly verified strongly reduced off-target activities of high fidelity compared with first-generation Cas9. Finally, the LATE assay was readily adapted to other cell types, namely clinically relevant human mesenchymal stromal cells (hMSCs) and retinal pigmented epithelial (RPE-1) cells. In conclusion, the LATE assay allows assessment of physiological adverse effects of the CRISPR/Cas system and might therefore be useful for preclinical safety studies.

INTRODUCTION

In 2011, clustered regularly interspaced short palindromic repeats (CRISPR)/CRISPR-associated protein 9 (Cas9) designer nucleases were first described for use in other than the original bacteria.[1] Soon thereafter, their DNA interference mechanism was precisely described by Doudna and Charpentier and collaborators.[2] Since then, the attention given to CRISPR/Cas has been rising exponentially. The system was quickly optimized toward application in eukaryotic and eventually human cells.[3–5] Meanwhile, CRISPR/Cas9 systems from various bacteria have been cloned, and many improvements have been introduced, e.g., to obtain high-fidelity enzymes that combine excellent efficiency with low off-target activity. The latest remarkable innovations were the introduction of base editors that allow direct correction of single-point mutations without the need of DNA strand breaks[6,7] and the invention of prime editing, a "search-and-replace" genome editing technology that facilitates a large variety of targeted genomic changes independent of cellular repair mechanisms. In fact, prime editing was shown to mediate targeted insertions, deletions, all 12 possible base-to-base conversions, and combinations thereof in human cells without requiring double-strand breaks (DSBs) or donor DNA templates.[8] Altogether, genome editing and particularly CRISPR/Cas9 have become a broadly used tool in many biotech fields but also in biomedicine.[9]

This fast progress has also led to the translation of first genome editing approaches toward clinical application, initially based on zinc-finger nucleases (ZFNs)[10] and transcription activator-like effector nucleases (TALENs),[11] but more recently also with CRISPR/Cas.[12] However, rapid translation into clinical testing has also raised some concerns. Indeed, rare unwanted effects of designer nucleases in general and CRISPR/Cas9 in particular might become relevant for large-scale application in human gene therapy, as previously observed with retroviral vectors.[13] Specifically, little is known about the actual impact of (1) immunogenicity of designer nucleases, (2) off-target cutting, and (3) the activation of the cellular repair response. In this regard, it is important to note that recent data even suggest that adverse effects might be more pronounced than anticipated. In fact, preexisting immune answers against Cas9 proteins of the most broadly used CRISPR/Cas9 systems from *Streptococcus aureus* and *Streptococcus pyogenes* were described in the vast majority of tested individuals.[14,15] Moreover, unexpectedly high rates of chromosomal aberrations after CRISPR/Cas9 treatment were found in recent studies.[16] In line with this, in studies with the CRISPR/Cas9 system

Received 28 April 2020; accepted 23 July 2021;
https://doi.org/10.1016/j.omtm.2021.07.004.

Correspondence: Boris Fehse, Research Department Cell and Gene Therapy, Department of Stem Cell Transplantation, University Medical Centre Hamburg-Eppendorf (UKE), Martinistr. 52, Hamburg 20246, Germany.
E-mail: fehse@uke.de
Correspondence: Dawid Głów, Research Department Cell and Gene Therapy, Department of Stem Cell Transplantation, University Medical Centre Hamburg-Eppendorf (UKE), Martinistr. 52, Hamburg 20246, Germany.
E-mail: d.glow@uke.de

This is an open access article under the CC BY license (http://creativecommons.org/licenses/by/4.0/).

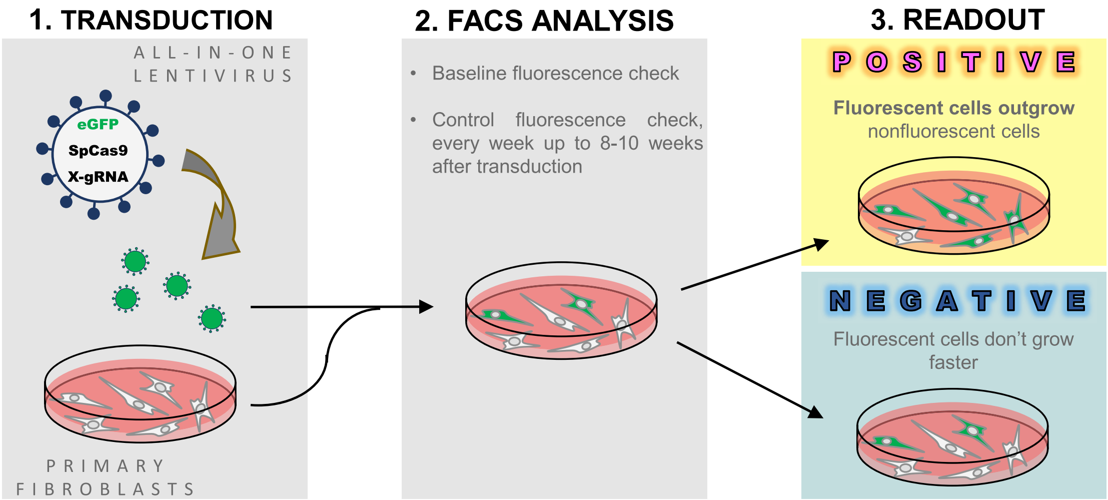

Figure 1. LATE assay principle

The principle of the LATE assay includes (1) transduction of primary human newborn foreskin fibroblasts (NUFFs) with all-in-one lentiviral vectors encoding fluorescent protein, designer nuclease (Cas9), and gRNA, (2) FC analysis of the cells for up to 10 weeks including baseline, and final check (3) readout, with increasing numbers of transduced cells as an indicator of a growth advantage acquired by the cells as a (side) effect of genome editing.

in rats and mice many off-target events were observed that had not been predicted by widely used bioinformatics algorithms.[17] Finally, a potential selection for p53-mutated cells as a result of genome editing was reported.[18] This is a worrying finding, since p53 mutations represent a hallmark of cancer and are present in almost all human malignancies.[19]

In view of the obvious risks associated with off-targets, many groups have investigated their actual frequencies. This has resulted not only in the establishment of various algorithms predicting likelihood of an off-target mutation at a given site (e.g., CRISPOR,[20] CHOP-CHOP,[21] and Cas-OFFinder[22]), but also in several unbiased, versatile techniques to profile genome-wide DSBs (e.g., GUIDE-seq,[23] Digenome-seq,[24] BLESS,[25] CIRCLE-seq,[26] Vivo [partially using CIRcLE-seq[27]], and qEva-CRISPR[28]). All the empirical methods rely on some form of high-throughput sequencing to detect mutations either in pre-selected regions or on a genome-wide scale. Next-generation sequencing (NGS), however, is a rather expensive method and requires advanced bioinformatics for detailed data analysis. This is particularly true for very rare events that require ultra-deep sequencing for detection. Moreover, all the above algorithms and methods are purely descriptive, i.e., they provide only information on locations and frequencies of the off-target mutations, whereas the much more relevant point—their potential consequences for cellular physiology—remains elusive. In order to minimize actual risks, it will be crucial to thoroughly investigate these potential side effects, thus ensuring well-grounded risk/chance assessments for genome editing-based clinical strategies. This holds particularly true for *in vivo* applications of genome editing and the problem of any hypothetical tumor-promoting side effects, as previously seen with integrating γ-retroviral vectors.[29] However, meaningful tests on potential adverse long-term effects of designer nucleases are still lacking.

To address this deficit, we have recently started development of the LATE assay (long-term adverse treatment effects identification assay). Deploying a worst-case scenario this assay aims at assessing the risk of growth-promoting off-target events based on the use of permanently genetically marked cells as targets of CRISPR/Cas9 delivery and sensitive readout methods including flow cytometry (FC) and digital PCR (ddPCR). In this proof-of-concept study we demonstrate the potential of the LATE assay to identify low-frequency growth-promoting off-target events and to compare off-target toxicity of different Cas9 proteins.

RESULTS

LATE assay principle: design and proof of concept

It was previously shown that *TP53* knockout in primary human newborn foreskin fibroblasts (NUFFs) leads to relative growth advantage resulting in positive selection and eventual outgrowth of affected clones.[30] We reasoned that this established growth advantage might be used for proving the concept of the envisioned principle of the LATE assay as depicted in Figure 1. To test the proposed assay design, we made use of an all-in-one lentiviral vector that co-expressed "classical" SpCas9, a *TP53*-targeting guide RNA (gRNA), and the eGFP marker ("LeGO-CC-p53"), where the gRNA targeted the previously described region[30] in exon 4 of *TP53* (Figure 2A, Table 1).

A sine qua non for the usefulness of the assay is the outgrowth of small numbers of mutated cells ensuring detection of rare events. To address this, we performed a limiting-dilution approach with decreasing amounts of LeGO-CC-p53 vector-containing supernatant (6 μL, 1.25 μL, 0.25 μL) applied to transduce NUFF cells. Initial transduction rates as measured by FC were 16.5% (calculated multiplicity of infection [MOI]: 0.2) and 3.2% (calculated MOI: 0.04) for the first two groups. At the same time, in the group transduced with the lowest

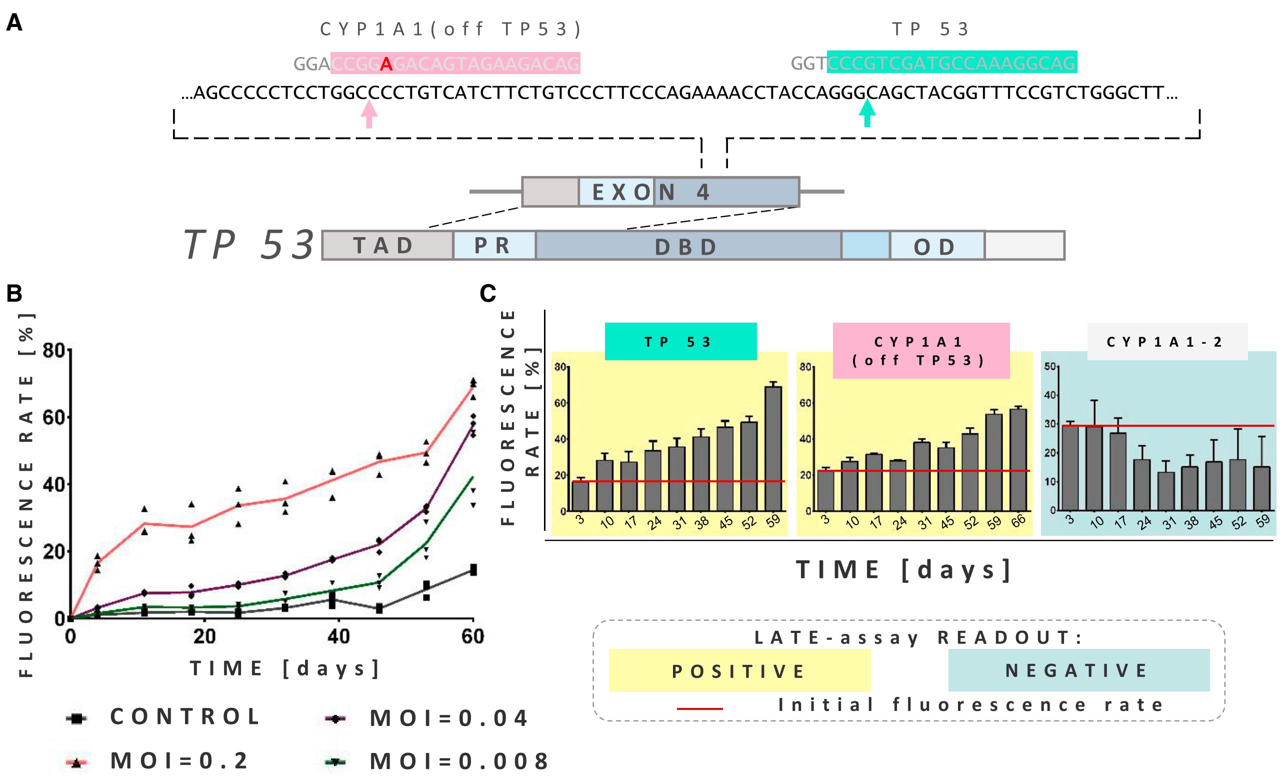

Figure 2. LATE assay is able to detect growth-promoting genome editing events

(A) Schematic representation of the *TP53* gene and two of the gRNA sequences applied to validate LATE assay (listed in Table 1). TP53 gRNA is fully complementary to the shown fragment of exon 4 of *TP53* gene, whereas CYP1A1(off TP53) gRNA contains a single mismatch to the *TP53* sequence (marked in red). Cas9 cleavage sites are marked with arrows. (B) Results of the FC analysis of the NUFF cells transduced with three different MOIs of all-in-one lentiviral particles encoding eGFP, Cas9, and TP53 gRNAs. (C) Comparison of the FC analysis of the NUFF cells transduced with all-in-one lentiviral particles encoding different gRNA sequences (TP53, CYP1A1(off TP53), or CYP1A1-2) marked with appropriate colors above the graphs. Red lines mark initial transduction rate. Based on LATE assay principles, positive and negative LATE results are marked with yellow and blue-gray colors, respectively (data are represented as mean ± SEM, n=3).

amount of vector (calculated MOI: 0.008), no transduced cells could be distinguished from the background fluorescence (control) based on eGFP expression (Figure 2B). Given the applied MOI, the expected transduction rate for this group would be ~0.5%. Cells were kept in culture and analyzed by FC every week for up to 9 weeks after transduction. As evident from Figure 2B, transduced cells had a strong growth advantage independent of the initial transduction rate. Based on the above transduction rate, the calculated number of transduced cells in the group transduced at the lowest MOI was below 150 (out of a total of 30,000 cells). This indicates that even with these low starting cell numbers the LATE assay has a high sensitivity, detecting growth-promoting events at frequencies below 0.5%.

To confirm that our observation was not related to donor-specific features, we repeated the LATE assay using the same LeGO-CC-p53 and NUFF cells from three different donors. In all three experiments the assay gave a positive output, showing acquired growth advantage of transduced cells (Figure S1). On the basis of these results, we defined the following principles for this first-generation LATE assay: (1) NUFF transduction with all-in-one lentiviral vectors, (2) checking baseline transduction rate and follow-up for up to 10 weeks by FC, and (3) positive readout, with increasing numbers of transduced cells as indicator of an acquired growth advantage in the process of genome editing (Figure 1).

Application of the LATE assay to detect off-target related adverse effects

Next, we asked whether the LATE assay will also be useful to detect growth advantages caused by unintended off-target knockout. To this end, we modeled the unwanted knockout of *TP53* as the result of the off-target activity of a CRISPR/Cas9-gRNA combination directed against a completely different target. Obviously, any off-target cutting of the *TP53* would be expected to occur at much lower frequencies compared with targeted *TP53* knockout, where a large percentage (if not all) of the transduced cells are predicted to harbor *TP53* mutations. Consequently, in the case of off-targeting *TP53*, only a small fraction of transduced (eGFP^{+}) cells will potentially obtain a growth advantage. Since the actual off-target activity of a given gRNA could not be predicted, we aimed at testing several gRNAs targeting different genes and having various degrees of similarity to the same

Table 1. List and specification of the gRNAs used and their alignment to *TP53* exon 4 sequence

gRNA name	Primary target	Targeting *TP53*	No. of MMs	Target sequence + PAM (corresponding TP53 sequence, MMs indicated)
TP53	*TP53*	yes	0	GACGGAAACCGTAGCTGCCC TGG
CYP1A1(off TP53)	*CYP1A1*	yes	1	GACAGAAGATGACAGAGGCC AGA (GACAGAAGATGACAGGGGCC AGG)
CYP1A1-2	*CYP1A1*	no	n.a.	GATTGGGCACATGCTGACCC TGG
CYP1A2(off TP53)	*CYP1A2*	yes	3	GGCAGAAGATGGCAGAGGCC AGG (GACAGAAGATGACAGGGGCC AGG)
CYP-BOTH	*CYP1A1 CYP1A2*	no	n.a.	ACCCGCACCTGGCACTGTCA AGG
GFOD(off TP53)	*GFOD1*	yes	2	GGAGCAGGAGCTGCTGGTGC AGG (GGTGTAGGAGCTGCTGGTGC AGG)
OBSL(off TP53)	*OBSL1*	yes	3	GCAGCCGTAGGTGCCCTGGT CGG (GAAACCGTAGCTGCCCTGGT AGG)
RFX(off TP53)	*RFX1*	yes	3	GAAGCCGGCGCTGCCCTGGT AGG (GAAACCGTAGCTGCCCTGGT AGG)
SOX(off TP53)	*SOX11*	yes	2	TGCAGGAGCTGCTGGTGCGG TGG (TGTAGGAGCTGCTGGTGCAG GGG)

MM, mismatches; n.a., not applicable.

exon as targeted by LeGO-CC-p53. We identified gRNAs matching our requirements of intentionally off-targeting *TP53* and picked six of them, containing one to three mismatches to the *TP53*-encoding sequence (Table 1).

These different gRNAs were next subjected to the LATE assay as described above. To this end, NUFF cells were transduced with all-in-one LeGO-CC vectors co-expressing Cas9 with each of the gRNAs. Transduced cells were kept in culture and analyzed by FC for up to 9 weeks. We aimed at initial transduction rates below 30% in order to deliver single vector copies to the majority of cells. At the same time, in our small-scale setting 30% transduction would result in a maximum of 9,000 transduced cells, which, for a *TP53* off-targeting probability of 1% would result in 90 cells with potential growth advantage. Indeed, over time a gradual increase in the proportion of eGFP$^+$ cells (positive readout) was observed in samples transduced with five of the six gRNAs off-targeting *TP53* and targeting *CYP1A1* (CYP1A1(off TP53)), *RFX1* (RFX(off TP53)), *OBSL1* (OBSL(off TP53)), *GFOD1* (GFOD(off TP53)), and *SOX11* (SOX(off TP53)) (Figure 2C; Figure S2). In contrast, there was no increase in numbers of transduced cells when the CYP1A2-targeting gRNA (CYP1A2(off TP53)) was used (Figure 2C). The negative result for the CYP1A2(off TP53) gRNA was confirmed with NUFF cells from 3 additional donors (Figure S1). Notably, this gRNA contains 3 mismatches to the *TP53*-encoding sequence, including a mismatch within 5 nucleotides of the 5′ end of the PAM sequence, i.e., within the so-called seed sequence. The latter is known to be most important for targeted DNA sequence recognition by the Cas9-gRNA complex. Therefore, the *CYP1A2*-specific gRNA had been expected to have the lowest probability of *TP53* off-targeting among all tested gRNA sequences.

In the next step, we asked whether the observed growth advantages of transduced cells might have been caused by a knockout of the primary targets rather than an off-target of the used gRNAs (Table 1). To do so, we designed gRNAs targeting either solely *CYP1A1* (CYP1A1-2) or a conserved region in both *CYP1A1* and *A2* (CYB-BOTH); both had no sequence similarity to *TP53* (Table 1; Figure S3). All-in-one LeGO-CC vectors were derived and used for the LATE assay with NUFF cells as described above. In both cases, no outgrowth of transduced cells was detected (Figure 2C; Figure S2), demonstrating that the growth advantages observed in LATE assays were obviously not caused by a knockout of *CYP1A1* or *CYP1A2*.

Positive LATE assay correlates with induced insertions/deletions in *TP53*

A positive readout in the LATE assay might theoretically also have been caused by reasons other than off-target *TP53* knockout, e.g., insertional mutagenesis.[31] To investigate the link between the positive readout of the LATE assay and (*de novo* generated) *TP53* indels, we applied gene-editing frequency digital PCR (GEF-dPCR), also referred to as drop-off assay, a technique developed in our laboratory.[32,33] GEF-dPCR allows quantification of insertion/deletion (indel) rates at the gRNA-binding site resulting from non-homologous end joining (NHEJ) during repair of the Cas9-mediated DSBs. In short, GEF-dPCR represents a modified "TaqMan" assay that utilizes two dual-labeled hydrolysis probes instead of one. One (here HEX labeled) probe binds to a region far from the gRNA recognition sequence, and thus its binding will expectedly not be influenced by the DNA repair process. In contrast, binding of the other (here FAM labeled) "drop-off" probe will be impaired by the presence of NHEJ-generated indels (probes and cleavage sites schematically presented in Figure 3A).

To test the approach, we first applied GEF-dPCR to enumerate indels at the *TP53* on-target site, i.e., after treatment with LeGO-CC-p53. We used three different amounts of vector supernatant (i.e., MOIs)

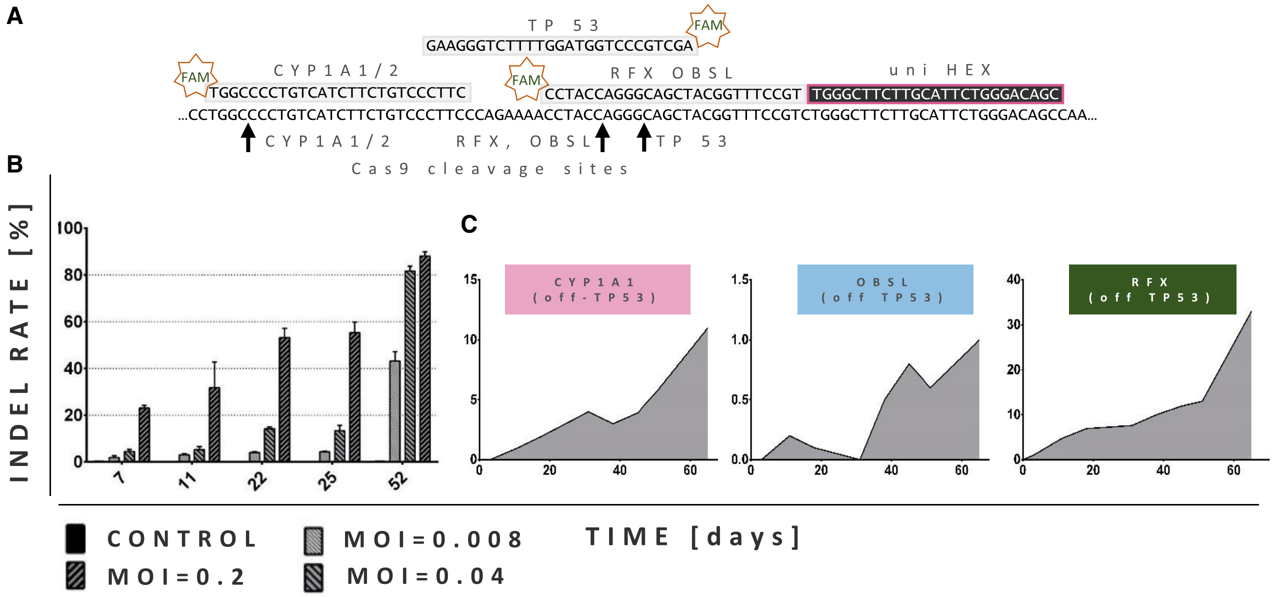

D

Sequence	Indel length [bp]	Frequency at day 7 [%]	Frequency at day 53 [%]	Change [%]
...GGCCCCTGTCATCTTCTGTCCCTTCCCAGAAAACCTACCAGGGCAGCTACGGTTTCCGTCTGGGCTT...	0 (WT)	68.21	6.82	-61.39
...GGCCCCTGTCATCTTCTGTCCCTTCCCAGAAAACCTACCAGGG-AGCTACGGTTTCCGTCTGGGCTT...	-1	15.84	52.71	+36.87
...GGCCCCTGTCATCTTCTGTCCCTTCCCAGAAAACCTACC-----AGCTACGGTTTCCGTCTGGGCTT...	-5	6.23	16.02	+9.79
...GGCCCCTGTCATCTTCTGTCCCTTCCCAGAAAACCTACCAGGG---------TTTCCGTCTGGGCTT...	-9	1.18	3.2	+2.02
...GGCCCCTGTCATCTTCTGTCCCTTCCCAGAAAAC------------CTACGGTTTCCGTCTGGGCTT...	-12	0.68	1.74	+1.06

Figure 3. Growth advantage gained by NUFF cells correlates with indel frequencies in TP53

(A) Graphical representation of the fragment of the *TP53* exon 4 and complementary FAM- and HEX-labeled probes used in GEF-dPCR. FAM-labeled probes were designed to bind positions to which Cas9 was targeted by gRNAs; thus, their binding to the *TP53* sequence depends on the absence of indels within their complementary region. HEX-labeled probe was designed to bind *TP53* sequence outside of the Cas9 cleavage sites. (B) On-target *TP53* indel rate (in exon 4) over time, measured by GEF-dPCR. Genomic DNA samples were obtained at the indicated time points from NUFF cells transduced with all-in-one lentiviral particles encoding eGFP, Cas9, and TP53-directed gRNA (LeGO-CC-p53) at three different MOIs (data are represented as mean ± SEM, n=3). (C) Off-target *TP53* indel rates (in exon 4) over time, measured by GEF-dPCR. Genomic DNA samples were obtained at the indicated time points from NUFF cells transduced with all-in-one lentiviral particles encoding gRNAs targeting Cas9 to *CYP1A1*, *OBSL1*, or *RFX1* and at the same time off-targeting *TP53* (Table 1). (D) Most frequent reads found in deep sequencing analysis of *TP53* exon 4 on days 7 and 53 after treatment with LeGO-CC-p53. Changes in the contribution of individual reads over time are indicated.

as in the previous experiment (see above). For each time point and MOI we isolated three samples of genomic DNA (gDNA), and for each gDNA isolation we pooled cells from two wells. In all of the measured samples we observed an increasing number of the *TP53* indels over time, starting from 1%–22% of the cells with indels at day 7 after transduction and peaking at 44%–85% cells after 52 days (Figure 3B). Notably, already at day 7 *TP53* indels were detectable for the lowest applied MOI of LeGO-CC-p53 (Figure 3B), even though initially there were essentially no transduced cells detectable by FC (see above, Figure 2B).

We moved on and applied GEF-dPCR to quantify indels at *TP53* off-target sites in cells transduced with LeGO-CC vectors encoding Cas9 and CYP1A1(off TP53), OBSL(off TP53), or RFX(off TP53) gRNAs. For this purpose, we used the same PCR primers and HEX-labeled probe and designed target-specific FAM-labeled "drop-off" probes (Figure 3A). We found that positive LATE assay readouts for all three gRNAs indeed correlated with increasing rates of *TP53* indels (Figure 3C; Figure S4). Notably, in the case of OBSL(off TP53) gRNA the first detectable *TP53* indel rates were as low as 0.2% (Figure 3C), but such low off-target activity became still detectable by outgrowth in the LATE assay. This observation underlines the sensitivity of the LATE assay in discovering even rare growth-promoting events. Importantly, we did not detect similar exon 4 *TP53* mutations/indels in any of the control samples.

Amplicon deep sequencing and RGB marking demonstrate clonal growth advantages in the LATE assay

The GEF-dPCR data indicated that the LATE assay allows detection of very rare events based on the outgrowths of potentially single cells. To confirm this assumption and validate GEF-dPCR data, we made use of two additional techniques: amplicon deep sequencing of the

targeted region in *TP53* exon 4 as well as red-green-blue (RGB) marking, a method for color-guided clonal cell tracking developed in our laboratory.[34,35]

First, we performed deep sequencing using genomic DNA isolated from NUFF cells 7 and 53 days after initial treatment with LeGO-CC-p53 (at MOI 0.2). At day 7, the indel rate measured by NGS (30.5%) was slightly higher than that estimated by GEF-dPCR (22%). This is not unexpected, since GEF-dPCR might be less susceptible to single-nucleotide losses or exchanges than NGS. In fact, among the three indels that occurred in >1% of the analyzed loci the deletion of one single cytidine (−1) was most frequent (16.13%) (Figure 3D). As expected, we observed a variety of different NHEJ-generated indels (Figure S5). On day 53 after transduction, only 6.84% of all NGS reads showed the wild-type sequence (Figure 3D), which was in accord with GEF-dPCR data (Figure 3B). Interestingly, the indels/clones most abundant already at day 7 remained predominant, with the variant containing the single cytidine deletion (−1) now representing 54% of all reads. The second most frequent *TP53* variant harbored a 5-nucleotide deletion (−5) and was uncovered in >16% of the analyzed loci (Figure 3D; Figure S5). Thus, these two frameshifting deletions were found together in >80% (compared to 22% at day 7) of all reads. These data underline the substantial growth advantage of NUFF cells harboring *TP53* frameshifting deletions.

To directly visualize potential clonal outgrowth, we prepared RGB-marked NUFF-derivative cells—fibroblasts marked with up to three (red-green-blue) fluorescence proteins (NUFF-RGB)—and transduced them with all-in-one LeGO-CC vectors encoding CYP1A1(off TP53) or TP53 gRNA. Transduced NUFF-RGB cells were submitted to the LATE assay.

As shown by FC (Figure S6), NUFF-RGB samples of both the CYP1A1(off TP53) and the TP53 gRNA groups showed evidence of clonal dominance as early as 25 days after transduction, whereas the control population remained largely polyclonal. This confirmed that the increasing proportions of eGFP-positive cells and *TP53* indel rates measured in the LATE assay were due to the outgrowth of limited numbers of cells gaining higher fitness.

Together our data obtained so far have proven that the LATE assay is able to detect clonal growth advantages resulting from tumor suppressor gene (TSG) loss of function caused by CRISPR/Cas9 on- as well as off-target activities in a very sensitive manner.

LATE assay can be applied to different cell types

For many purposes, NUFF cells might not be the relevant indicator cell type. To address this limitation, we next tested the versatility of the LATE assay. To this end, we applied it to two further cell types: (1) human mesenchymal stromal cells (hMSCs) and (2) human telomerase reverse transcriptase (h-TERT)-immortalized primary retinal pigmented epithelial (RPE-1) cells. hMSCs are of great interest, particularly in regenerative medicine, thanks to their multi-lineage differentiation capacity and their immune-regulatory properties. Their potential in regenerative medicine was recently reviewed in detail by Han and collaborators.[36] In line with that work, genome editing is a promising approach to generate tailored hMSCs for different clinical applications. RPE-1 cells combine genetic features of primary cells with the cell line's ability to divide continuously *in vitro* and might therefore be useful to develop standardized assays to detect potential adverse effects of genome editing in these cells.

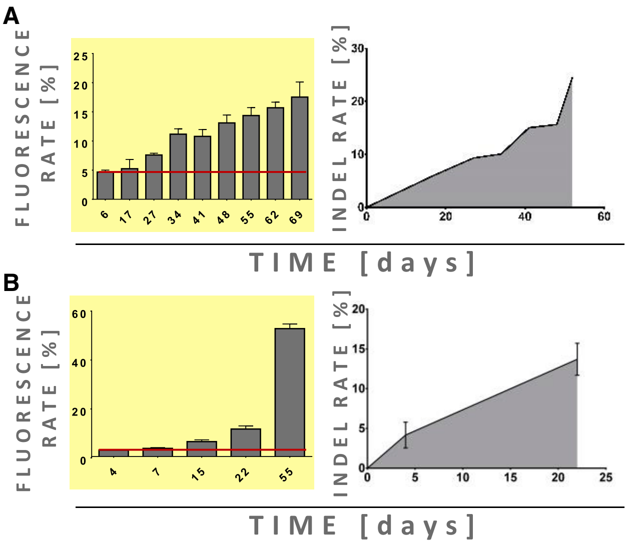

Figure 4. LATE assay can be applied to hMSCs and to h-TERT-immortalized cells

Results of the LATE assay in hMSCs (A) and RPE-1 cells (B). Left: Percentages of eGFP+ MSCs/RPE-1 cells over time after transduction with all-in-one lentiviral particle LeGO-CC-p53 encoding eGFP, Cas9, and TP53-directed gRNAs. Red line marks initial transduction rate. Right: Relative numbers of indels in exon 4 of *TP53* over time as measured by GEF-dPCR. For each DNA isolation, cells from three wells from a 12-well plate were pooled (hMSCs) or measured separately (RPE-1 cells) (data are represented as the pooled sample result or by mean ± SEM, n=3).

To provide proof of principle, we again made use of all-in-one LeGO-CC-p53 vectors. However, in view of the significantly different transduction efficiencies of hMSCs and RPE-1 cells with our vesicular stomatitis virus glycoprotein (VSV-G)-pseudotyped vectors we had to apply much higher MOIs of LeGO-CC-p53, i.e., concentrated vector preparations to transduce hMSCs and lower MOIs to transduce RPE-1 cells. Again, in both cases, we observed an induced growth advantage of eGFP+ cells manifesting in their slow but permanent outgrowth (Figures 4A and 4B, left). As for NUFF, we next asked whether this increase was due to an increasing number of cells harboring indels within *TP53*. To do so, we again applied GEF-dPCR to enumerate cells with mutated *TP53* at the gRNA on-target site (Figures 4A and 4B, right). As expected, the observed overgrowth of eGFP+ cells in both cases was correlated with increasing rates of *TP53* indels.

These data indicate that the principle of the LATE assay can readily be adapted to different types of cultured and primary cells. This will be of

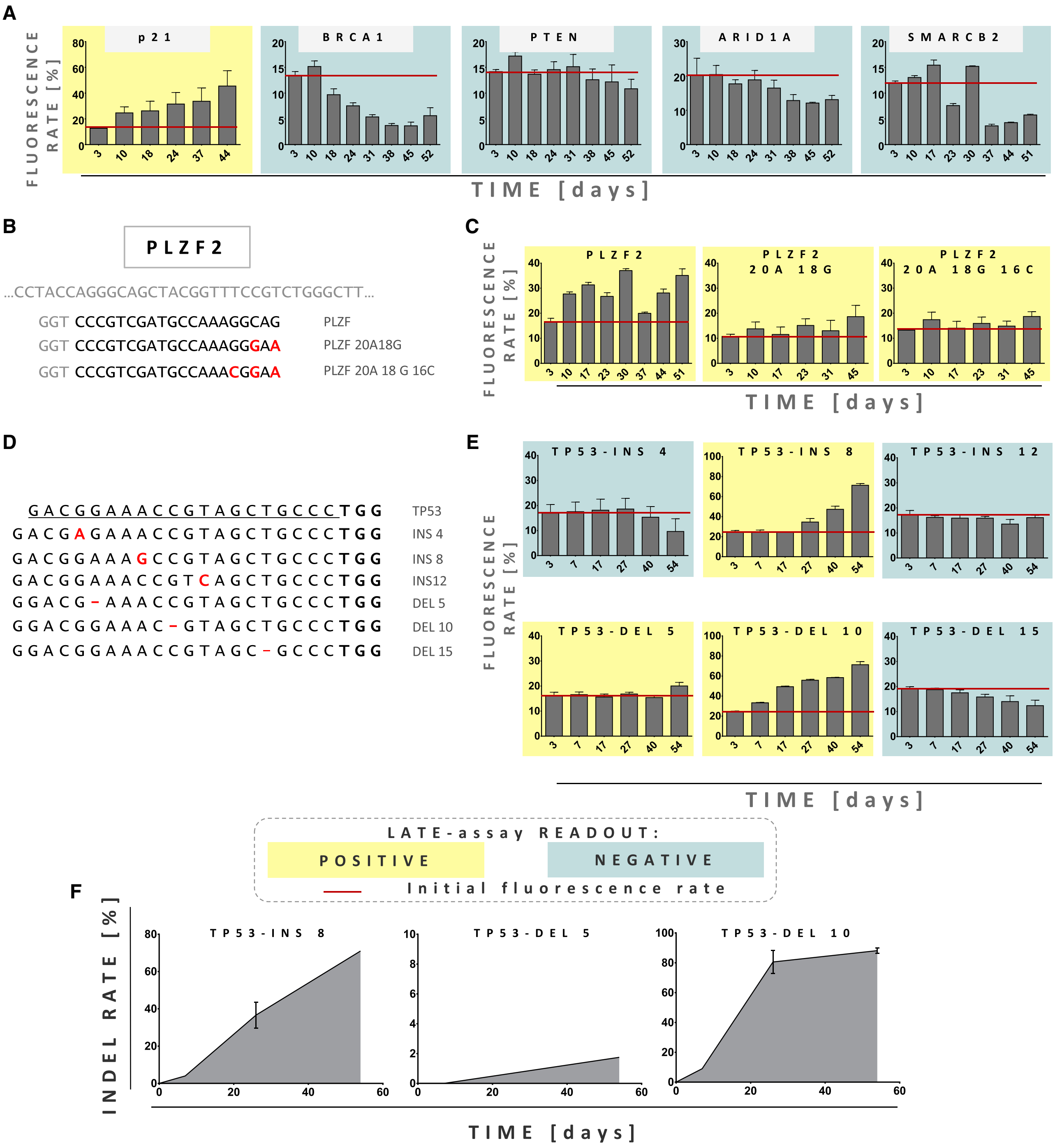

Figure 5. LATE assay is able to detect growth-promoting events in different TSGs

(A) FC analysis of NUFF cells transduced with all-in-one lentiviral particles encoding eGFP, Cas9, and gRNAs targeting different functional classes of TSG: *p21*, *BRCA1*, *PTEN*, *ARID1A*, and *SMARCB2*. Red lines mark initial transduction rate. Based on LATE assay principles, positive and negative LATE results are marked with yellow and blue-gray colors, respectively (data are represented as mean ± SEM, n = 3). (B) Graphical representation of the fragment of the *PLZF* and gRNAs used in the experiment. Mismatches are marked in red. (C) FC analysis of the NUFF cells transduced with all-in-one lentiviral particles encoding eGFP, Cas9, and gRNAs presented in (B). Red lines mark initial transduction rate. Based on LATE assay principles, positive and negative LATE results are marked with yellow and blue-gray colors, respectively (data are

(legend continued on next page)

potential relevance, since side effects of genome editing might be cell type specific.

LATE assay detects growth-accelerating events for gRNAs not targeting *TP53*

With a sole focus on *TP53*, the usefulness of the assay would be limited, since off-targeting of the *TP53* gene could be largely avoided by carefully designing gRNAs. That is why we extended our preliminary work to other TSGs of different functional classes. These included genes encoding proteins involved in DNA repair (*BRCA1*) as well as genes involved in epigenetic modifications (*ARID1A*, *SMARC2B*), transcription (*PLZF*), and cell death/cell cycle control (*PTEN*, *p21*). Again, we exemplarily applied the LATE assay to NUFF cells, in which the chosen TSGs were targeted. Importantly, we found different targets that gave positive readouts in the LATE assay, particularly *p21* and *PLZF* (Figures 5A and 5C, respectively). That prompted us to test whether the application of Cas9 in combination with gRNAs containing mismatches to *PLZF* will result in measurable growth advantage of transduced cells. We designed these gRNAs to harbor either 2 or 3 mismatches at the 5′ end of the gRNA (Figure 5B). In both cases, the LATE assay gave positive results, proving its broad applicability. In summary, these results clearly demonstrate that the LATE assay is not limited to *TP53* but can detect off-targets in a variety of genes.

Still, it could be argued that gRNAs off-targeting TSGs (or proto-oncogenes) would readily be predicted by bioinformatics tools and therefore could be excluded. Importantly, recent work demonstrated that gRNAs might also bind to (off-target) sequences containing single-nucleotide indels compared to their actual target sequence.[37,38] Cleavage of such targets by Cas nucleases requires bulge formation, a possibility obviously not foreseen by the algorithms employed by commonly used off-target predicting tools that apparently fail to identify those potential off-target sequences.[37,38] We therefore tested empirically whether indel-containing *TP53*-specific gRNAs will still promote a growth of transduced NUFF cells and thus result in a positive readout of the LATE assay. To do so, we designed 6 gRNA spacers with single-base indels compared to the *TP53* sequence. To conserve the 20-nt length of the spacer sequence, we balanced deletions with the addition of a single G base at the 5′ end of gRNA (Figure 5D). We transduced NUFF cells with all-in-one LeGO-CC vectors as above and observed growth advantage (= positive LATE readout) in 3 out of 6 cases (Figure 5E). The LATE readout was again confirmed by GEF-dPCR (Figure 5F). Interestingly, 2 of the 3 gRNAs (INS8 and DEL10) were not predicted to off-target *TP53* by CRISPOR, one of the most frequently used tools for off-target prediction. In contrast, Off-Spotter listed *TP53* as a potential off-target but indicated 5 mismatches, thus suggesting an extremely low likelihood for off-target activity. The only tested online tool that indicated a high *TP53* off-targeting probability for the indel-containing gRNAs was the search tool offered by Integrated DNA Technologies (IDT). In fact, IDT's online tool was able to precisely show the type and position of differences between designed spacers and *TP53* sequences.

LATE assay facilitates assessment of Cas9 specificity

Early after the introduction of CRISPR/Cas9, the comparatively high off-target activity of the new genome editing tools became apparent.[39–41] Since then, significant efforts have been made to decrease the probability of unwanted off-target cutting. Those improvements were directed toward both compounds of CRISPR/Cas9 system, nucleic acid and protein,[42,43] but the Cas9 nuclease has become the preferred target to increase on-target specificity. In fact, SpCas9 has been subjected to rational design for structural modifications as well as random mutagenesis, and multiple Cas9 variants of increased specificity have been engineered, e.g., K855ACas9, eSpCas9,[44] HF-Cas9,[45] HypaCas9,[46] and SniperCas9.[47] A single alanine substitution of the positively charged residue (K855A) was described to lower Cas9 off-target activity.[44] Almost no detectable off-target activity (within the used detection limit) was found for a Cas9 variant with quadruple substitutions (Cas9-HF1-N497A/R661A/Q695A/Q926A).[45] We wanted to test whether our LATE assay is able to discriminate between different Cas9 variants with regard to their actual specificities on the functional level. To do so, we performed targeted mutagenesis to generate the above mentioned SpCas9 K855A, with a single amino acid substitution, and eSpCas9 1.0 harboring alanine substitution of three positively charged residues (K810A/K1003A/R1060A). We replaced the classical Cas9 by each one of the improved Cas9 variants in several all-in-one LeGO-CC vectors combining them with the TP53, CYP1A1(off TP53), and CYP1A1-2 gRNAs. Using those vectors, we transduced NUFF cells and performed the standard LATE assay as above.

First, we analyzed the nuclease activity of SpCas9 K855A by on-targeting *TP53*. As shown in Figure 6A, at similar initial transduction rates the outgrowth kinetics of eGFP-positive cells were comparable for SpCas9 K855A and wt Cas9-treated cells. We again confirmed the presence of indels in the targeted region of *TP53* by GEF-dPCR and deep amplicon sequencing. As with wt Cas9, both techniques confirmed increasing numbers of cells harboring indels in *TP53* over time: With GEF-dPCR the maximum indel frequency reached ~65% at day 52 (Figure 6A), whereas almost 80% of NGS reads contained indels at day 55 post transduction (Figure S6). Again, we found a variety of NHEJ-generated indels; the most abundant *TP53* indel variants were single cytidine deletion (−1) (change from 8% to 28%) and deletion of five base pairs (change from 0.9% to 19.5%) (Figure 6B; Figure S7).

represented as mean ± SEM, n = 3). (D) Alignment of the TP53-specific gRNAs and the gRNA variants containing single-nucleotide indels. Indels are marked with red color. (E) FC analysis of the NUFF cells transduced with all-in-one lentiviral particles encoding eGFP, Cas9, and gRNAs presented in (D). Based on LATE assay principles, positive and negative LATE results are marked with yellow and blue-gray colors, respectively (data are represented as mean ± SEM, n = 3). Average of the initial transduction rate of each sample is marked with a red line. (F) Off-target *TP53* indel rates (in exon 4) over time, measured by GEF-dPCR. Genomic DNA samples were obtained at the indicated time points from NUFF cells transduced with all-in-one lentiviral particles encoding gRNAs/RNPs targeting *TP53 (data are represented as mean ± SEM, n=3)*.

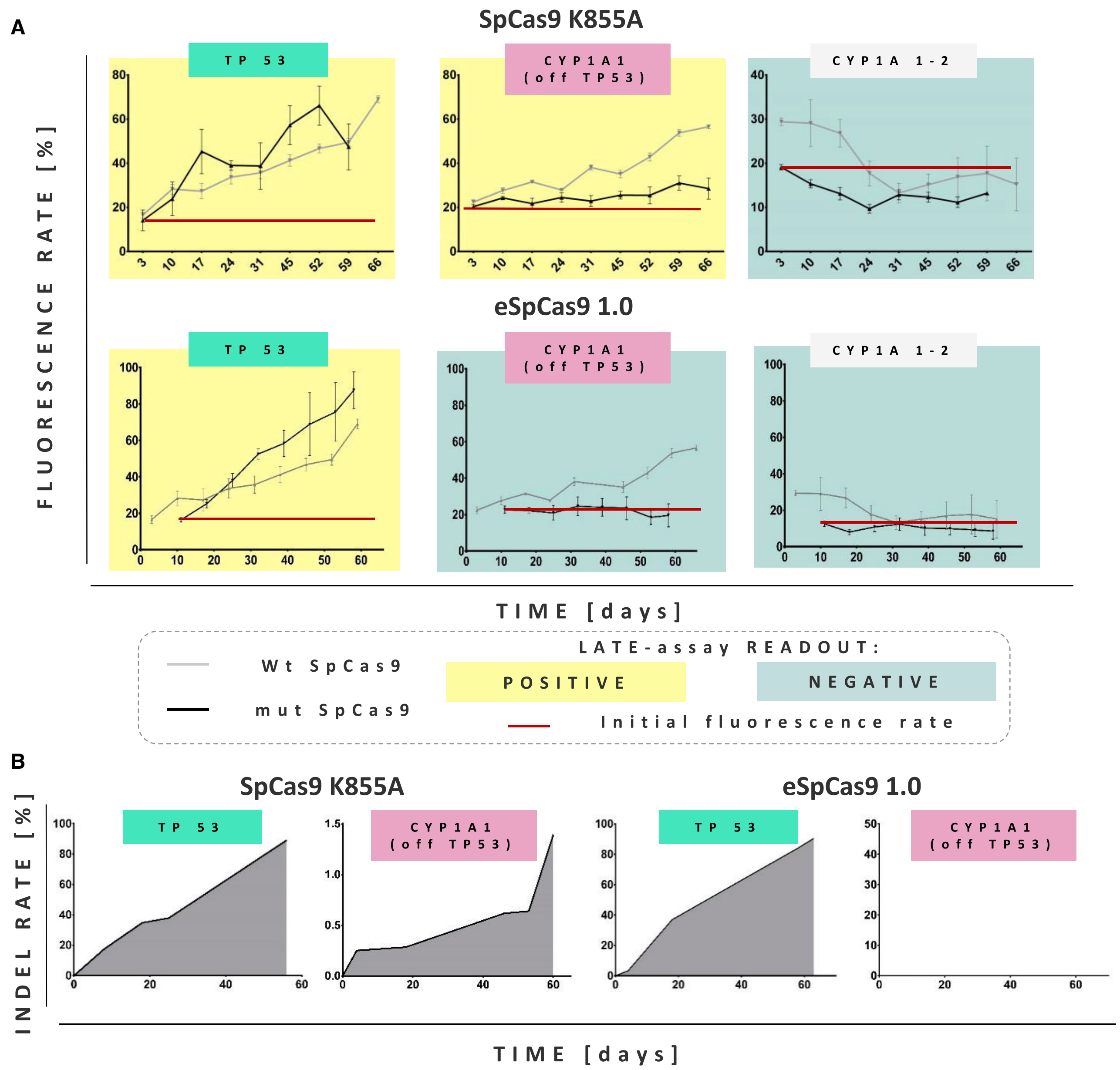

Figure 6. LATE assay distinguishes SpCas9 variants with different fidelities

(A) Comparison of the FC analysis of NUFF cells transduced with all-in-one LeGO-CC particles encoding wild-type (wt) SpCas9 (gray) or one of two mutated (mut) variants of SpCas9 (black). gRNAs were directed against *TP53* and *CYP1A1* (off-targeting and not off-targeting *TP53*) as indicated above the graphs. Red lines mark initial transduction rates for vectors encoding mutated SpCas9 variants. Following LATE principles, assay positivity and negativity are indicated by yellow and blue-gray colors, respectively (data are represented as mean ± SEM, n=3). (B) Relative numbers of indels over time as measured by GEF-dPCR after on-targeting (TP53) and off-targeting (CYP1A1(off TP53)) exon 4 of *TP53*. Data are shown separately for both SpCas9 mutants. For each DNA isolation, cells from three wells of a 24-well plate were pooled.

Interestingly, when SpCas9 K855A was used with CYP1A1(off TP53) gRNA, the proportion of the eGFP$^+$ cells was growing much slower compared to the NUFF cells treated with the same gRNA and wt Cas9 (Figure 6A). The LATE assay eventually became "positive"; the slower and smaller outgrowth indicated lower initial off-target rates and was thus consistent with the predicted improved SpCas9 K855A specificity. Also as expected, no measurable growth advantage of eGFP$^+$ cells was observed when CYP1A1-2 gRNA was applied (Figure 6A). To confirm our phenotypic observations on the molecular level, we again used GEF-dPCR to quantify indel numbers within the off-targeted region of *TP53* (exon 4). As evident from Figure 6B, there was an excellent concordance between the delayed outgrowth of

eGFP$^+$ cells and the corresponding late increase in *TP53* indel rates with SpCas9 K855A.

In a second set of experiments, we performed the same analyses for eSpCas9 1.0, reported to have even further improved specificity. Indeed, off-targeting *TP53* with CYP1A1(off TP53) gRNA we did not observe an outgrowth of eGFP$^+$ cells in the LATE assay, whereas *TP53* on-targeting was at least as efficient as with wt Cas9 (Figure 6A). Accordingly, we did not detect any indel formation in the off-targeted region of *TP53* by GEF-dPCR (Figure 6B). As expected, the LATE assay was negative with CYP1A1-2 gRNA (Figure 6A). These data confirm the excellent specificity of eSpCas9 1.0 and clearly prove the usefulness of the LATE assay to test specificity of different nucleases using a relevant functional readout.

DISCUSSION

Genome editing has revolutionized many areas of basic bioresearch as well as applied biotechnology. As a next big step, broad implementation of genome editing techniques in clinical gene therapy has been suggested. However, unwanted side effects, e.g., due to off-target cutting, often not relevant or easily excluded in research, might represent a definitive risk in human applications, where huge cell numbers will be modified and consequences of side effects might develop over decades. In fact, malignant transformation mediated by insertional mutagenesis had long been considered an extremely unlikely event in retroviral gene therapy, until it was first observed in a murine study[48] and shortly thereafter in the SCID-X1 trial in Paris.[13]

Therefore, suitable assays are required that allow thorough testing of novel genome editing techniques regarding their potential adverse effects. Whereas a large variety of techniques was introduced to predict and empirically detect off-targets of designer nuclease,[20–29] the obtained genetic information can in the best case only indirectly predict possible functional consequences of off-targeting.

To address this problem, we designed and established a novel cellular assay referred to as the LATE assay. In this work we have provided proof of concept demonstrating that the LATE assay facilitates detection of the *functional* impact of Cas9 off-target cutting. To do so, we first designed gRNAs targeting relevant genes and simultaneously off-targeting *TP53*, a crucial TSG. We were able to demonstrate that the LATE assay is indeed able to detect growth-promoting genome editing events within *TP53* as well as other TSGs, namely *p21* and *PLZF*. Our finding provides, to our knowledge, the first demonstration of an assay that allows discovery of physiologically adverse effects of the CRISPR/Cas system, specifically gain of growth advantage, one of the major tumorigenicity hallmarks.[19]

In our proof-of-concept study, we used flow cytometry as the main readout to directly visualize the growth advantage of cells that underwent genome editing. Subsequently, we confirmed that the observed growth advantage was indeed related to on- and off-target activity of Cas9 in conjunction with the tested gRNAs on the molecular level. To do so, we used a dedicated digital-PCR technique (GEF-dPCR)[32,33] as well as deep amplicon sequencing for direct indel detection in the target region. Finally, we showed that clonal outgrowth can be directly visualized by RGB marking.[34,35]

To establish the principle of the LATE assay, we tested the different Cas9-gRNA combinations on 30,000 NUFF cells per single well (in triplicates). Using this low cell number, we were still able to detect *functionally relevant* off-target events at frequencies below 0.5%. It is safe to propose that the LATE assay can easily be upscaled to much larger cell numbers, which expectedly will result in higher sensitivities. However, even the current sensitivity is comparable with NGS-based methods, bounded by the actual error rate of NGS for indels (~0.1%). Considering the easy analysis and the relatively low costs (particularly as compared to deep sequencing required for similar sensitivity) the LATE assay has also clear advantages from a practical point of view.

Given the universal readout, the LATE assay can be used with any type of designer nuclease, including different CRISPR/Cas variants. In fact, we have shown that our assay clearly distinguished functionally relevant off-target activities of wt Cas9 and recently developed high-fidelity variants. In addition, we provided evidence that the LATE assay can readily be adapted to other cell types, particularly highly relevant primary hMSCs. Thus, the LATE assay can be employed to verify cell type-specific probabilities and impact of off-target activities of a given designer nuclease.

Current limitations of the LATE assay include the long time for readout and the low cell numbers used. Obviously, with starting cell numbers of 30,000 and transduction rates of 20%, the maximal sensitivity would be limited to ~1 off-target event in 2,000 cells (considering Poisson distribution). However, as noted above and supported by our preliminary data (not shown), the assay should be readily scalable to ensure higher sensitivities. In the presented proof-of-principle setting we focused on off-targets in well-known TSGs such as *TP53* and the easy FC-based readout. At the same time, we do not see any reason why the assay should not work with other growth-promoting events as well. Moreover, the assay might readily be adapted to more subtle changes in the clonal composition by including sensitive readouts, such as DNA barcoding.[49,50]

Finally, one might argue that the tested situation is too far from reality, since no one would use a gRNA off-targeting any established TSG, proto-oncogene, or regulatory sequence thereof. In this regard it was important to observe that the LATE assay was able to detect TP53-mediated adverse effects for gRNAs that were not labeled with a "red flag" by commonly used online tools. In fact, two gRNAs conferring *TP53* knockout were not found to off-target this gene by the popular CRISPOR tool, whereas Off-Spotter listed *TP53* as an unlikely off-target containing 5 mismatches. In contrast, the IDT online tool correctly predicted the *TP53* off targets. These data confirmed recent findings by Lin et al.[38] and Jones et al.[37] that Cas9 does accept single-nucleotide indels in the target region and highlight the importance of adapting the algorithms for off-target identification to include single-nucleotide indels in all prediction tools.

In conclusion, we propose that the LATE assay presented here might fill the gap as an easy, fast, and cheap technology for testing unwanted adverse effects of a given genome editing strategy on cell growth regulation. The assay is complementary to genome-wide off-target detection methods based on NGS and should always be used in conjunction. Finally, the LATE assay provides a handy tool to test specificity of genome editing instruments, e.g., different Cas9 nucleases.

MATERIALS AND METHODS

Cells, cell lines, and cell culture

Primary fibroblasts, HEK293T (ATCC CRL-3216), RPE-1 cells (ATCC CRL-4000), and their derivatives were cultured in Dulbecco's modified Eagle's medium (DMEM Glutamax, Life Technologies) supplemented with 10% fetal calf serum (FCS) (5% for RPE-1 cells), L-glutamine (2 mM), penicillin (100 U/mL), and streptomycin (100 μg/mL). hMSCs were cultured in DMEM (Life Technologies) supplemented with 10% non-heat-inactivated FCS and L-glutamine (2 mM).

Cell culture was performed under standard conditions (37°C, 100% relative humidity, 5% CO_2). Cell culture material was purchased from Corning (Corning, NY, USA), Greiner Bio One (Frickenhausen, Germany), and Sarstedt (Nümbrecht, Germany).

Molecular cloning

All-in-one CRISPR/Cas9 LeGO ("LeGO-CC") vectors[51] were generated by cloning human codon-optimized *Streptococcus pyogenes* Cas9 gene (SpCas9) containing Nuclear Localization Signal and chimeric gRNA scaffold under hU6 promotor from pX330 (3, Addgene #42230), a kind gift from the Feng Zhang lab, into LeGO-iG2 (Addgene #27341) and LeGO-iC2 (Addgene #27345) vectors.[52,53] To generate constructs that express the gRNAs of choice, the respective sequences with addition of ACC at the 5′ end of the leading strand, followed by G if necessary (required for polymerase III dependent transcription) and AAC at 5′ end of complementary strand, were synthetized. ACC/AAC were added to allow for cloning into the SapI cloning site of LeGO-CC-iC2 or LeGO-CC-iG2 vectors.

To obtain the K855A and e1.0 (K810A/K1003A/R1060A) Cas9 variants, suitable primers were designed to introduce the desired mutations by PCR with Q5 DNA polymerase (NEB, Ipswich, MA, USA). The resulting linear PCR products were digested with DpnI for 1 h at 37°C and subsequently circularized with T4 ligase (Thermo Fisher Scientific, Waltham, MA, USA) for 2 h at 16°C in a blunt-end ligation. Ligation, transformation, and plasmid preps followed common protocols.

Primer sequences used in this project are shown in Table S1.

Lentiviral vector production and cell transduction

Described all-in-one LeGO-CC vectors were used to obtain third-generation lentiviral particles that were produced in accordance with our standard protocols.[52] VSV-G-encoding plasmid (phCMV-VSV-G) was used for viral pseudotyping.[52] In brief, 5×10^6 cells were seeded and after overnight culture were transfected with appropriate plasmids. Medium was exchanged after 6 h, and the viral supernatant was harvested and filtered 24 h later. If indicated, all-in-one LeGO-CC vectors were concentrated by centrifugation (4°C, 16 h, 8,000 × *g*).

To transduce primary fibroblasts with lentiviral supernatant, 3×10^4 cells were plated in 500 μL of medium in a 24-well plate. Two hours later viral supernatants and polybrene (8 μg/mL) were added (in triplicates). After 24 h medium was exchanged to standard growth medium. To check the initial transduction rate, cells were analyzed ~72–96 h after transduction by FC. Subsequently, cells were analyzed at different time points as indicated in Results.

To transduce hMSCs with lentiviral supernatant, 5×10^4 cells were plated in 1 mL of medium supplemented with 20 mM HEPES in a 12-well plate. Two hours later concentrated viral supernatants and polybrene (8 μg/mL) were added in triplicates. Further steps did not differ from the protocol used for transducing fibroblasts.

RGB marking of NUFF cells

RGB marking of cells for clonal tracking was performed as described previously.[34,35] For the labeling of primary human NUFFs, 50,000 cells were plated per well of a 12-well cell culture plate in 1 mL of medium. RGB marking was carried out with concentrated stocks of three VSV-G-pseudotyped LeGO vectors, each encoding a fluorescent protein of one of the basic colors under control of an EF1α promoter, linked to a puromycin resistance by a 2A sequence: LeGO-EF1a-B2-Puro+ (encoding mTagBFP, blue), LeGO-EF1a-V2-Puro+ (encoding Venus, green), and LeGO-EF1a-C2-Puro+ (encoding mCherry, red).[53] Functional titers were in the range of 3.5×10^8 to 6.2×10^8/mL, titrated on 293T cells. An absolute MOI of ~3 (MOI 1 per color) was applied for optimal color distribution within the RGB-marked cells. Selection of transduced cells was done by addition of 1 μg/mL puromycin.

Flow cytometry

Cells from 12/24-well plates were dissociated with 400/200 μL of 0.5% trypsin-EDTA. Cells were transferred to FC tubes and centrifuged for 5 min at 310 × *g*. Cell pellets were resuspended in 250 μL of PBS and subjected to FC analysis, performed on LSRFortessa or CantoII (both BD Biosciences) at the Cytometry and Cell Sorting Core Unit of the UMC Hamburg-Eppendorf. Data were analyzed with BD FACSDiva (BD Biosciences) or FlowJo (FlowJo). Initially, cells were gated based on (1) SSC-A/FSC-A, (2) FSC-A/FSC-H, (3) FSC-A/eGFP-A parameters. In such cases, the fluorescence rate of control samples is presented. To reduce autofluorescence signal, we measured an additional parameter and changed the gating strategy to (1) SSC-A/FSC-A, (2) FSC-A/FSC-H, (3) mCherry-A/eGFP-A. After FC data were obtained, cells were collected for genomic DNA purification.

Digital PCR

Fibroblasts and hMSCs were harvested, and genomic DNA was purified with the DNeasy Blood & Tissue Kit (QIAGEN) and quantified

with a NanoDrop Spectrophotometer (Thermo Fisher Scientific). ddPCRs were designed, prepared, and carried out essentially as described in our published protocol,[32,33] but on a Naica Crystal Digital PCR system (Stilla Technologies, Villejuif, France). In short, PCR mixtures were assembled with 1× PerfeCTa Multiplex qPCR ToughMix (Quanta Biosciences, Gaithersburg, MD, USA), 100 nM fluorescein (Sigma, St. Louis, MO, USA), 1 μM dPCR p53 forward primer and 1 μM dPCR p53 reverse primer for amplification of the target sequence, 250 nM p53-p53 HEX reference probe, and 40–80 ng of genomic DNA. 250 nM of FAM-drop-off probe was added corresponding to the cleavage site that was to be analyzed (FAM CYP1-p53 for CYP1A1(off TP53) sgRNA off-target cleavage site and FAM p53-p53 for TP53 on-target cleavage site). PCR reactions were loaded onto Sapphire or Opal chips (Stilla Technologies), compartmentalized into 2D monolayers of droplet partitions, and amplified with the Naica Geode instrument. Cycling conditions were step 1: 95°C for 3 min; step 2: 95°C for 10 s; step 3: 61°C for 1 min; repeat steps 2 and 3 45 times. Chips containing the generated droplets were imaged with the Naica Prism3 reader (Stilla Technologies), and fluorescent data were analyzed with Crystal Miner software (Stilla Technologies). Negative and positive droplets were discriminated by manual thresholding according to the wt controls included in each individual experiment.

To analyze TP53 off-target of wt Cas9 targeting OBSL1, RFX1, and CYP1A1 the digital PCR (dPCR) reaction mixture was prepared with the following: 1× ddPCR supermix for probes (no dUTP; Bio-Rad) and the same amounts of the primers and DNA as in the case of the reactions prepared to analyze on the Naica system. DG8 Cartridges (Bio-Rad) were filled with ddPCR reaction mixtures and Droplet Generation Oil for Probes (Bio-Rad). Droplets were generated with a QX100 Droplet Generator (Bio-Rad) according to the manufacturer's instructions and then transferred into a 96-well PCR plate for PCR using a T100 Thermal Cycler (Bio-Rad). We used the same PCR program as in the Naica system. Data were analyzed with QuantaSoft software (Bio-Rad). Negatives and positives droplets were discriminated by manual thresholding according to the non-edited controls included in each individual experiment.

Deep sequencing

To prepare the sequencing libraries, fibroblasts were harvested at the indicated time point, and genomic DNA was purified with the DNeasy Blood & Tissue Kit (QIAGEN) and quantified with a NanoDrop Spectrophotometer (Thermo Fisher Scientific). The genomic region flanking the CRISPR target site was amplified. To do so, 60–80 ng of the genomic DNA was submitted to PCR (Q5 polymerase, NEB) that simultaneously added Illumina adapters to the amplicons (primer sequences in Table S1). Cycling conditions were step 1: 98°C for 2 min; step 2: 98°C for 10 s; step 3: 69°C for 20 s; step 4: 72°C for 20 s; repeat steps 2–4 5 times; step 5: 98°C for 10 s; step 6: 72°C for 30 s; repeat steps 5 and 6 30 times. PCR products were purified with the PCR Purification Kit (QIAGEN), following the manufacturer's protocol. Purified DNA samples were quantified with Qubit (Thermo Fisher Scientific) and adjusted to 20 ng/μL. NGS of the prepared libraries and data analysis service were performed by GENEWIZ on Illumina devices. A minimal amount of 50,000 reads for each sample were obtained. Received data were analyzed with the web-based tool CRISPResso2.[54]

Statistical analysis

Datasets shown as bar graphs represent the average of three independent experiments, with error bars indicating standard deviation (SD), if not specified otherwise. Statistical significance was determined with a two-tailed, homoscedastic Student's t test.

SUPPLEMENTAL INFORMATION

Supplemental information can be found online at https://doi.org/10.1016/j.omtm.2021.07.004.

ACKNOWLEDGMENTS

The authors gratefully acknowledge the professional technical assistance of Tanja Sonntag and Almut Uhde. We would like to thank Pia Houdek from the Department of Dermatology and Venerology of the University Medical Centre Hamburg-Eppendorf (UKE) for providing NUFF cells for our research. The authors appreciate the help of the employees of UKE's "Cytometry and Cell Sorting" Core Unit. This work has been supported by the Deutsche Forschungsgemeinschaft (DFG) within SFB841 (S02).

AUTHOR CONTRIBUTIONS

D.G., K.R., and B.F. designed the study, S.M., I.G.R., L.M.A., and D.G. performed the experiments, D.G. prepared the figures and wrote the first draft of the manuscript, and B.F. wrote the final version. All authors have approved the final version of the manuscript.

DECLARATION OF INTERESTS

The authors declare no competing interests.

REFERENCES

1. Sapranauskas, R., Gasiunas, G., Fremaux, C., Barrangou, R., Horvath, P., and Siksnys, V. (2011). The Streptococcus thermophilus CRISPR/Cas system provides immunity in Escherichia coli. Nucleic Acids Res. *39*, 9275–9282.

2. Jinek, M., Chylinski, K., Fonfara, I., Hauer, M., Doudna, J.A., and Charpentier, E. (2012). A programmable dual-RNA-guided DNA endonuclease in adaptive bacterial immunity. Science *337*, 816–821.

3. Cong, L., Ran, F.A., Cox, D., Lin, S., Barretto, R., Habib, N., Hsu, P.D., Wu, X., Jiang, W., Marraffini, L.A., and Zhang, F. (2013). Multiplex genome engineering using CRISPR/Cas systems. Science *339*, 819–823.

4. Mali, P., Yang, L., Esvelt, K.M., Aach, J., Guell, M., DiCarlo, J.E., Norville, J.E., and Church, G.M. (2013). RNA-guided human genome engineering via Cas9. Science *339*, 823–826.

5. Jinek, M., East, A., Cheng, A., Lin, S., Ma, E., and Doudna, J. (2013). RNA-programmed genome editing in human cells. eLife *2*, e00471.

6. Komor, A.C., Kim, Y.B., Packer, M.S., Zuris, J.A., and Liu, D.R. (2016). Programmable editing of a target base in genomic DNA without double-stranded DNA cleavage. Nature *533*, 420–424.

7. Nishida, K., Arazoe, T., Yachie, N., Banno, S., Kakimoto, M., Tabata, M., Mochizuki, M., Miyabe, A., Araki, M., Hara, K.Y., et al. (2016). Targeted nucleotide editing using hybrid prokaryotic and vertebrate adaptive immune systems. Science *353*, aaf8729.

8. Anzalone, A.V., Randolph, P.B., Davis, J.R., Sousa, A.A., Koblan, L.W., Levy, J.M., Chen, P.J., Wilson, C., Newby, G.A., Raguram, A., and Liu, D.R. (2019). Search-and-replace genome editing without double-strand breaks or donor DNA. Nature *576*, 149–157.

9. Wang, H., La Russa, M., and Qi, L.S. (2016). CRISPR/Cas9 in Genome Editing and Beyond. Annu. Rev. Biochem. *85*, 227–264.

10. Tebas, P., Stein, D., Tang, W.W., Frank, I., Wang, S.Q., Lee, G., Spratt, S.K., Surosky, R.T., Giedlin, M.A., Nichol, G., et al. (2014). Gene editing of CCR5 in autologous CD4 T cells of persons infected with HIV. N. Engl. J. Med. *370*, 901–910.

11. Qasim, W., Zhan, H., Samarasinghe, S., Adams, S., Amrolia, P., Stafford, S., Butler, K., Rivat, C., Wright, G., Somana, K., et al. (2017). Molecular remission of infant B-ALL after infusion of universal TALEN gene-edited CAR T cells. Sci. Transl. Med. *9*, eaaj2013, 28123068.

12. Xu, L., Wang, J., Liu, Y., Xie, L., Su, B., Mou, D., Wang, L., Liu, T., Wang, X., Zhang, B., et al. (2019). CRISPR-Edited Stem Cells in a Patient with HIV and Acute Lymphocytic Leukemia. N Engl J Med. *381*, 1240–1247. https://doi.org/10.1056/NEJMoa1817426.

13. Hacein-Bey-Abina, S., Von Kalle, C., Schmidt, M., McCormack, M.P., Wulffraat, N., Leboulch, P., Lim, A., Osborne, C.S., Pawliuk, R., Morillon, E., et al. (2003). LMO2-associated clonal T cell proliferation in two patients after gene therapy for SCID-X1. Science *302*, 415–419.

14. Charlesworth, C.T., Deshpande, P.S., Dever, D.P., Camarena, J., Lemgart, V.T., Cromer, M.K., Vakulskas, C.A., Collingwood, M.A., Zhang, L., Bode, N.M., et al. (2019). Identification of preexisting adaptive immunity to Cas9 proteins in humans. Nat. Med. *25*, 249–254.

15. Crudele, J.M., and Chamberlain, J.S. (2018). Cas9 immunity creates challenges for CRISPR gene editing therapies. Nat. Commun. *9*, 3497.

16. Kosicki, M., Tomberg, K., and Bradley, A. (2018). Repair of double-strand breaks induced by CRISPR-Cas9 leads to large deletions and complex rearrangements. Nat. Biotechnol. *36*, 765–771.

17. Anderson, K.R., Haeussler, M., Watanabe, C., Janakiraman, V., Lund, J., Modrusan, Z., Stinson, J., Bei, Q., Buechler, A., Yu, C., et al. (2018). CRISPR off-target analysis in genetically engineered rats and mice. Nat. Methods *15*, 512–514.

18. Haapaniemi, E., Botla, S., Persson, J., Schmierer, B., and Taipale, J. (2018). CRISPR-Cas9 genome editing induces a p53-mediated DNA damage response. Nat. Med. *24*, 927–930.

19. Petitjean, A., Mathe, E., Kato, S., Ishioka, C., Tavtigian, S.V., Hainaut, P., and Olivier, M. (2007). Impact of mutant p53 functional properties on *TP53* mutation patterns and tumor phenotype: lessons from recent developments in the IARC *TP53* database. Hum. Mutat. *28*, 622–629.

20. Haeussler, M., Schönig, K., Eckert, H., Eschstruth, A., Mianné, J., Renaud, J.B., Schneider-Maunoury, S., Shkumatava, A., Teboul, L., Kent, J., et al. (2016). Evaluation of off-target and on-target scoring algorithms and integration into the guide RNA selection tool CRISPOR. Genome Biol. *17*, 148.

21. Labun, K., Montague, T.G., Krause, M., Torres Cleuren, Y.N., Tjeldnes, H., and Valen, E. (2019). CHOPCHOP v3: expanding the CRISPR web toolbox beyond genome editing. Nucleic Acids Res. *47* (W1), W171–W174.

22. Bae, S., Park, J., and Kim, J.-S. (2014). Cas-OFFinder: a fast and versatile algorithm that searches for potential off-target sites of Cas9 RNA-guided endonucleases. Bioinformatics *30*, 1473–1475.

23. Tsai, S.Q., Zheng, Z., Nguyen, N.T., Liebers, M., Topkar, V.V., Thapar, V., Wyvekens, N., Khayter, C., Iafrate, A.J., Le, L.P., et al. (2015). GUIDE-seq enables genome-wide profiling of off-target cleavage by CRISPR-Cas nucleases. Nat. Biotechnol. *33*, 187–197.

24. Kim, D., Bae, S., Park, J., Kim, E., Kim, S., Yu, H.R., Hwang, J., Kim, J.I., and Kim, J.S. (2015). Digenome-seq: genome-wide profiling of CRISPR-Cas9 off-target effects in human cells. Nat. Methods *12*, 237–243, 1, 243.

25. Yan, W.X., Mirzazadeh, R., Garnerone, S., Scott, D., Schneider, M.W., Kallas, T., Custodio, J., Wernersson, E., Li, Y., Gao, L., et al. (2017). BLISS is a versatile and quantitative method for genome-wide profiling of DNA double-strand breaks. Nat. Commun. *8*, 15058.

26. Tsai, S.Q., Nguyen, N.T., Malagon-Lopez, J., Topkar, V.V., Aryee, M.J., and Joung, J.K. (2017). CIRCLE-seq: a highly sensitive in vitro screen for genome-wide CRISPR-Cas9 nuclease off-targets. Nat. Methods *14*, 607–614.

27. Akcakaya, P., Bobbin, M.L., Guo, J.A., Malagon-Lopez, J., Clement, K., Garcia, S.P., Fellows, M.D., Porritt, M.J., Firth, M.A., Carreras, A., et al. (2018). In vivo CRISPR editing with no detectable genome-wide off-target mutations. Nature *561*, 416–419.

28. Dabrowska, M., Czubak, K., Juzwa, W., Krzyzosiak, W.J., Olejniczak, M., and Kozlowski, P. (2018). qEva-CRISPR: a method for quantitative evaluation of CRISPR/Cas-mediated genome editing in target and off-target sites. Nucleic Acids Res. *46*, e101.

29. Baum, C., Kustikova, O., Modlich, U., Li, Z., and Fehse, B. (2006). Mutagenesis and oncogenesis by chromosomal insertion of gene transfer vectors. Hum. Gene Ther. *17*, 253–263.

30. Knopp, Y., Geis, F.K., Heckl, D., Horn, S., Neumann, T., Kuehle, J., Meyer, J., Fehse, B., Baum, C., Morgan, M., et al. (2018). Transient retrovirus-based CRISPR/Cas9 all-in-one particles for efficient, targeted gene knockout. Mol. Ther. Nucleic Acids *13*, 256–274.

31. Modlich, U., Bohne, J., Schmidt, M., von Kalle, C., Knöss, S., Schambach, A., and Baum, C. (2006). Cell-culture assays reveal the importance of retroviral vector design for insertional genotoxicity. Blood *108*, 2545–2553.

32. Mock, U., Machowicz, R., Hauber, I., Horn, S., Abramowski, P., Berdien, B., Hauber, J., and Fehse, B. (2015). mRNA transfection of a novel TAL effector nuclease (TALEN) facilitates efficient knockout of HIV co-receptor CCR5. Nucleic Acids Res. *43*, 5560–5571.

33. Mock, U., Hauber, I., and Fehse, B. (2016). Digital PCR to assess gene-editing frequencies (GEF-dPCR) mediated by designer nucleases. Nat. Protoc. *11*, 598–615.

34. Weber, K., Thomaschewski, M., Warlich, M., Volz, T., Cornils, K., Niebuhr, B., Täger, M., Lütgehetmann, M., Pollok, J.M., Stocking, C., et al. (2011). RGB marking facilitates multicolor clonal cell tracking. Nat. Med. *17*, 504–509.

35. Weber, K., Thomaschewski, M., Benten, D., and Fehse, B. (2012). RGB marking with lentiviral vectors for multicolor clonal cell tracking. Nat. Protoc. *7*, 839–849.

36. Han, Y., Li, X., Zhang, Y., Han, Y., Chang, F., and Ding, J. (2019). Mesenchymal Stem Cells for Regenerative Medicine. Cells *8*, 886.

37. Jones, S.K., Jr., Hawkins, J.A., Johnson, N.V., Jung, C., Hu, K., Rybarski, J.R., Chen, J.S., Doudna, J.A., Press, W.H., and Finkelstein, I.J. (2021). Massively parallel kinetic profiling of natural and engineered CRISPR nucleases. Nat. Biotechnol. *39*, 84–93.

38. Lin, Y., Cradick, T.J., Brown, M.T., Deshmukh, H., Ranjan, P., Sarode, N., Wile, B.M., Vertino, P.M., Stewart, F.J., and Bao, G. (2014). CRISPR/Cas9 systems have off-target activity with insertions or deletions between target DNA and guide RNA sequences. Nucleic Acids Res. *42*, 7473–7485.

39. Fu, Y., Foden, J.A., Khayter, C., Maeder, M.L., Reyon, D., Joung, J.K., and Sander, J.D. (2013). High-frequency off-target mutagenesis induced by CRISPR-Cas nucleases in human cells. Nat. Biotechnol. *31*, 822–826.

40. Cradick, T.J., Fine, E.J., Antico, C.J., and Bao, G. (2013). CRISPR/Cas9 systems targeting β-globin and *CCR5* genes have substantial off-target activity. Nucleic Acids Res. *41*, 9584–9592.

41. Hsu, P.D., Scott, D.A., Weinstein, J.A., Ran, F.A., Konermann, S., Agarwala, V., Li, Y., Fine, E.J., Wu, X., Shalem, O., et al. (2013). DNA targeting specificity of RNA-guided Cas9 nucleases. Nat. Biotechnol. *31*, 827–832.

42. Moon, S.B., Kim, D.Y., Ko, J.H., and Kim, Y.S. (2019). Recent advances in the CRISPR genome editing tool set. Exp. Mol. Med. *51*, 1–11.

43. Moon, S.B., Kim, D.Y., Ko, J.H., Kim, J.S., and Kim, Y.S. (2019). Improving CRISPR genome editing by engineering guide RNAs. Trends Biotechnol. *37*, 870–881.

44. Slaymaker, I.M., Gao, L., Zetsche, B., Scott, D.A., Yan, W.X., and Zhang, F. (2016). Rationally engineered Cas9 nucleases with improved specificity. Science *351*, 84–88.

45. Kleinstiver, B.P., Pattanayak, V., Prew, M.S., Tsai, S.Q., Nguyen, N.T., Zheng, Z., and Joung, J.K. (2016). High-fidelity CRISPR-Cas9 nucleases with no detectable genome-wide off-target effects. Nature *529*, 490–495.

46. Chen, J.S., Dagdas, Y.S., Kleinstiver, B.P., Welch, M.M., Sousa, A.A., Harrington, L.B., Sternberg, S.H., Joung, J.K., Yildiz, A., and Doudna, J.A. (2017). Enhanced proofreading governs CRISPR-Cas9 targeting accuracy. Nature *550*, 407–410.

47. Lee, J.K., Jeong, E., Lee, J., Jung, M., Shin, E., Kim, Y.H., Lee, K., Jung, I., Kim, D., Kim, S., and Kim, J.S. (2018). Directed evolution of CRISPR-Cas9 to increase its specificity. Nat. Commun. *9*, 3048.

48. Li, Z., Düllmann, J., Schiedlmeier, B., Schmidt, M., von Kalle, C., Meyer, J., Forster, M., Stocking, C., Wahlers, A., Frank, O., et al. (2002). Murine leukemia induced by retroviral gene marking. Science *296*, 497.

49. Cornils, K., Thielecke, L., Hüser, S., Forgber, M., Thomaschewski, M., Kleist, N., Hussein, K., Riecken, K., Volz, T., Gerdes, S., et al. (2014). Multiplexing clonality: combining RGB marking and genetic barcoding. Nucleic Acids Res. *42*, e56.

50. Selich, A., Daudert, J., Hass, R., Philipp, F., von Kaisenberg, C., Paul, G., Cornils, K., Fehse, B., Rittinghausen, S., Schambach, A., and Rothe, M. (2016). Massive Clonal Selection and Transiently Contributing Clones During Expansion of Mesenchymal Stem Cell Cultures Revealed by Lentiviral RGB-Barcode Technology. Stem Cells Transl. Med. *5*, 591–601.

51. Timin, A.S., Muslimov, A.R., Lepik, K.V., Epifanovskaya, O.S., Shakirova, A.I., Mock, U., Riecken, K., Okilova, M.V., Sergeev, V.S., Afanasyev, B.V., et al. (2018). Efficient gene editing via non-viral delivery of CRISPR-Cas9 system using polymeric and hybrid microcarriers. Nanomedicine (Lond.) *14*, 97–108.

52. Weber, K., Bartsch, U., Stocking, C., and Fehse, B. (2008). A multicolor panel of novel lentiviral "gene ontology" (LeGO) vectors for functional gene analysis. Mol. Ther. *16*, 698–706.

53. Weber, K., Mock, U., Petrowitz, B., Bartsch, U., and Fehse, B. (2010). Lentiviral gene ontology (LeGO) vectors equipped with novel drug-selectable fluorescent proteins: new building blocks for cell marking and multi-gene analysis. Gene Ther. *17*, 511–520.

54. Clement, K., Rees, H., Canver, M.C., Gehrke, J.M., Farouni, R., Hsu, J.Y., Cole, M.A., Liu, D.R., Joung, J.K., Bauer, D.E., and Pinello, L. (2019). CRISPResso2 provides accurate and rapid genome editing sequence analysis. Nat. Biotechnol. *37*, 224–226.

OMTM, Volume *22*

Supplemental information

LATE–a novel sensitive cell-based assay for the study of CRISPR/Cas9-related long-term adverse treatment effects

Dawid Głów, Simon Meyer, Irene García Roldán, Lara Marie Akingunsade, Kristoffer Riecken, and Boris Fehse

Table S1. Oligonucleotides and oligonucleotide probes used in the study.

R NAME	SEQUENCE	REMARKS
Fspcas9K855A	GCGGTGCTGACCAGAAG	PCR primers for introduction K855A substitution into Cas9 coding sequence
Rspcas9K855A	GTTGTCGATGGAGTCGTCC	
Fspcas9K810A	GCGCTGTACCTGTACTACCTGCAG	PCR primers for introduction K810A substitution into Cas9 coding sequence
Rspcas9K810A	CTCGTTCTGCAGCTGGGTG	
Fspcas9K1003A	GCGCTGGAAAGCGAGTTC	PCR primers for introduction K1003A substitution into Cas9 coding sequence
Rspcas9K1003A	AGGGTACTTTTTGATCAGGGCG	
Fspcas9R1060A	GCGCCTCTGATCGAGACAAACG	PCR primers for introduction R1060A substitution into Cas9 coding sequence
Rspcas9R1060A	CTTCCGGATCTCGCCGTTGG	
Fcyp1a1-2	ACCGATTGGGCACATGCTGACCC	Contain CYP1A1 targeting protospacer sequence
Rcyp1a1-2	AACGGGTCAGCATGTGCCCAATC	
FcypBOTH	ACCGACCCGCACCTGGCACTGTCA	Contain CYP1A1 and CYP1A2 targeting protospacer sequence
RcypBOTH	AACTGACAGTGCCAGGTGCGGGTC	
fCYP1A1	ACCGACAGAAGATGACAGAGGCC	Contain CYP1A1 targeting protospacer sequence
rCYP1A1	AACGGCCTCTGTCATCTTCTGTC	
fSOX11	ACCGTGCAGGAGCTGCTGGTGCGG	Contain SOX1 targeting protospacer sequence
rSOX11	AACCCGCACCAGCAGCTCCTGCAC	
fGFOD1	ACCGGAGCAGGAGCTGCTGGTGC	Contain GFOD1 targeting protospacer sequence
rGFOD1	AACGCACCAGCAGCTCCTGCTCC	
fCYP1A2	ACCGGCAGAAGATGGCAGAGGCC	Contain CYP1A2 targeting protospacer sequence
rCYP1A2	AACGGCCTCTGCCATCTTCTGCC	
fRFX1	ACCGAAGCCGGCGCTGCCCTGGT	Contain RFX1 targeting protospacer sequence
rRFX1	AACACCAGGGCAGCGCCGGCTTC	
fOBSL1	ACCGCAGCCGTAGGTGCCCTGGT	Contain OBSL1 targeting protospacer sequence
rOBSL1	AACACCAGGGCACCTACGGCTGC	
fTP53	ACCGACGGAAACCGTAGCTGCCC	Contain TP53 targeting protospacer sequence
rTP53	AACGGGCAGCTACGGTTTCCGTC	
fARID1A	ACCGCCGGACCTGAAGAACTCGAA	Contain ARID1A targeting protospacer sequence
rARID1A	AACTTCGAGTTCTTCAGGTCCGGCGGT	

fSMARCB2	ACCGACGGCGAGTTCTACATGAT	Contain SMARCB2 targeting protospacer sequence		
rSMARCB2	AACATCATGTAGAACTCGCCGTCGGT			
fPTEN	ACCGCTAACGATCTCTTTGATGA	Contain PTEN targeting protospacer sequence		
rPTEN	AACTCATCAAAGAGATCGTTAGC			
fBRCA1	ACCGCTTGTGCTGACTTACCAGAT	Contain BRCA1 targeting protospacer sequence		
rBRCA1	AACATCTGGTAAGTCAGCACAAGC			
fp21	ACCGCGCGACTGTGATGCGCTAA	Contain p21 targeting protospacer sequence		
rp21	AACTTAGCGCATCACAGTCGCGC			
fPLZF2	ACCGTGTGCAAGGCCAACCAGATG	Contain PLZF2 targeting protospacer sequence		
rPLZF2	AACCATCTGGTTGGCCTTGCACAC			
fPLZF2-2MM	ACCGAGAGCAAGGCCAACCAGATG	Contain PLZF2 off-targeting (2MM) protospacer sequence		
rPLZF2-2MM	AACCATCTGGTTGGCCTTGCTCTC			
fPLZF2-3MM	ACCGAGAGGAAGGCCAACCAGATG	Contain PLZF2 off-targeting (3MM) protospacer sequence		
rPLZF2-3MM	AACCATCTGGTTGGCCTTCCTCTC			
		5' modification	3' modification	REMARKS
FddP53	CCTGCACCAGCAGCTCCTAC	-	-	Used for GEF-dPCR TP53 exon-4 mutation
RddP53	CAACTGACCGTGCAAGTCACA	-	-	
HexP53	TGGGCTTCTTGCATTCTGGGACAGC	HEX	BHQ	
FamRFXobsl-p53	CCTACCAGGGCAGCTACGGTTTCCGT	FAM	BHQ	
FamP53-p53	AGCTGCCCTGGTAGGTTTTCTGGGAAG	FAM	BHQ	
FamCYP1-p53	TGGCCCCTGTCATCTTCTGTCCCTTC	FAM	BHQ	
AMPLICON NGS PRIMERS				
FngsTP53ex4	ACACTCTTTCCCTACACGACGCTCTTCCGATCTCCTGCACCAGCAGCTCCTAC			
FngsTP53ex4	GACTGGAGTTCAGACGTGTGCTCTTCCGATCTCAACTGACCGTGCAAGTCACA			

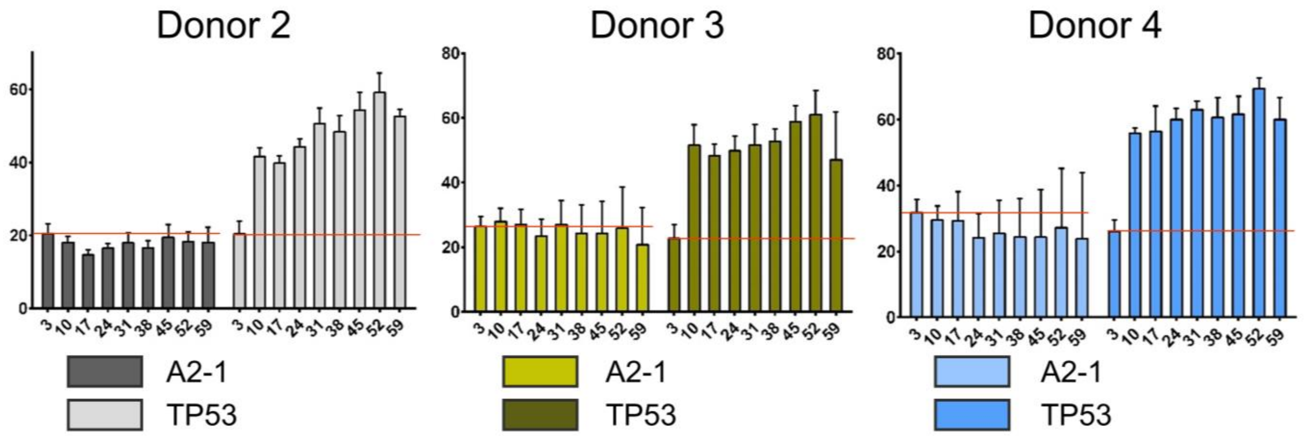

Supplementary Figure S1. LATE-assay readout is not depended on donor specific features. Graph shows Results of the FC analysis of the NUFF from three different donors, transduced with all-in-one lentiviral particles encoding eGFP, wtCas9 and TP53 or A2-1 gRNAs. Red lines mark initial transduction rate. Increase in GFP+ cells can be observed in case of all of the cells transduced with TP53 targeting lentiviruses.

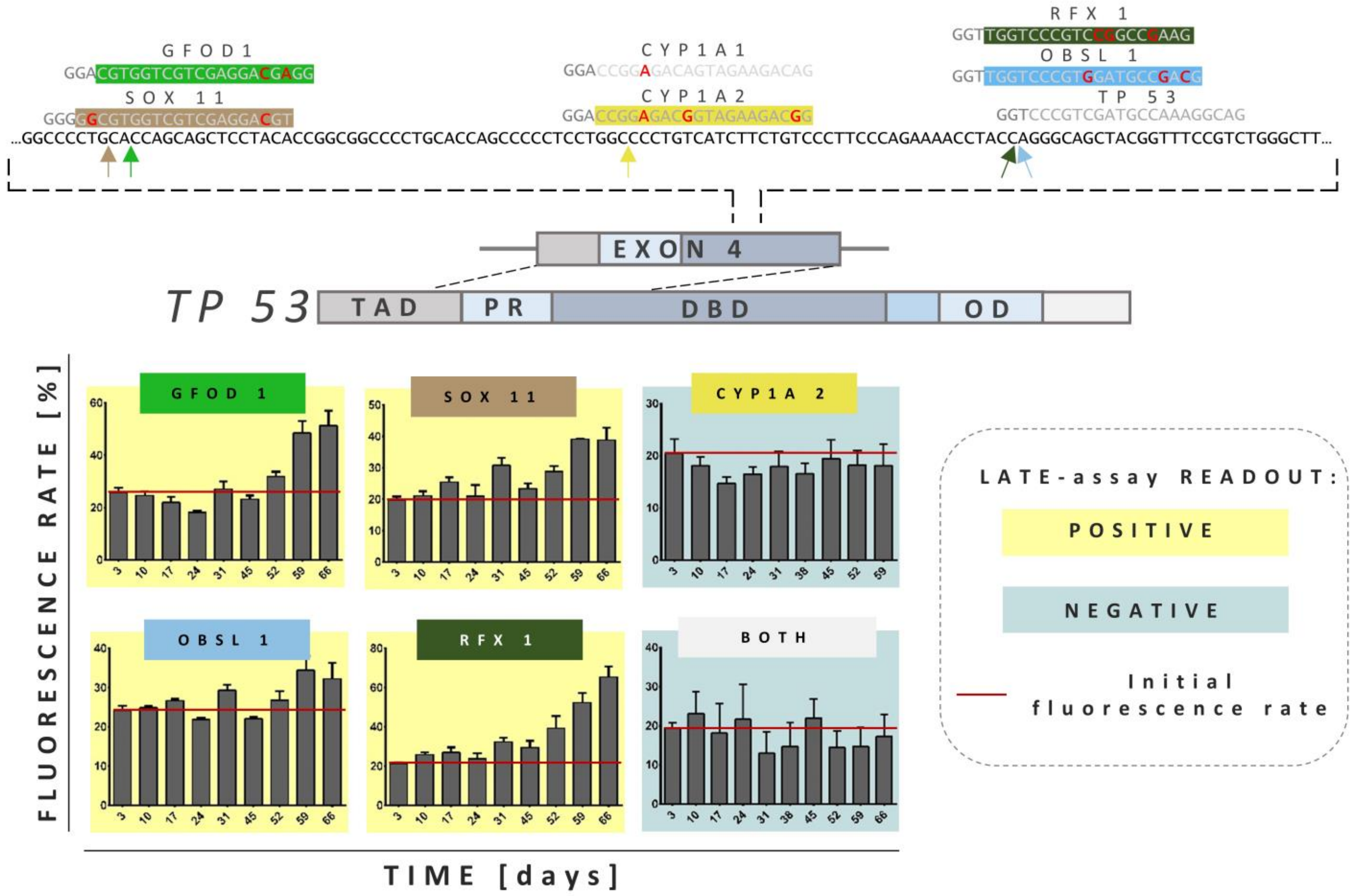

Supplementary Figure S2. LATE-assay is able to detect growth-promoting genome editing events. A) Schematic representation of the TP53 gene and part of the spacer sequences applied to validate LATE-assay (listed in Table 1). Mismatches to the TP53 sequence were marked in red. Cas9 cleavage sites are marked with arrows which colors indicate adequate spacers. B) Comparison of the FC analysis of the NUFF cells transduced with all-in-one lentiviral particles encoding different spacer sequences (OBSL, RFX or A2-1, CYP-BOTH, SOX, GFOD) marked with adequate colors above the graphs. Red lines mark initial transduction rate. Based on the LATE-assay principles, positive and negative LATE results were marked with yellow and blue-grey colors respectively. n=3

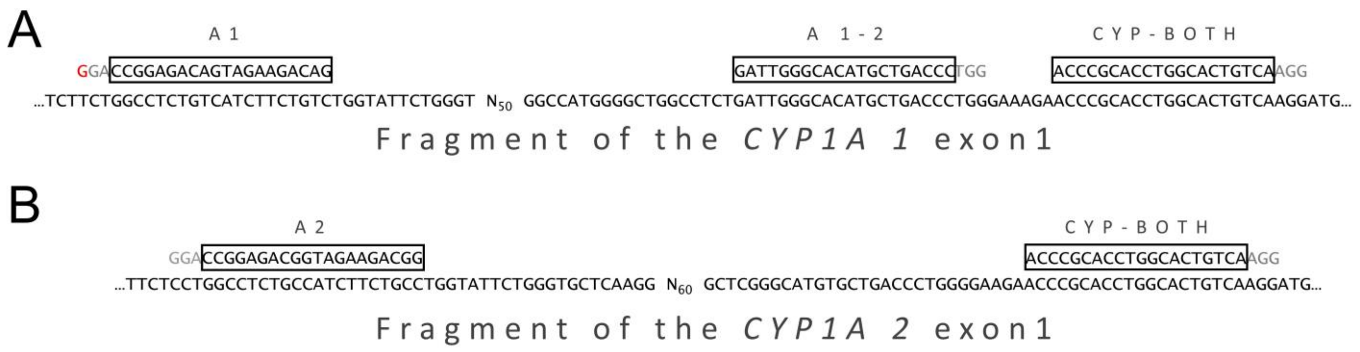

Supplementary Figure S3. Schematic representation of the CYP1A1 and CYP1A2 genes fragments to which spacer sequences were designed.

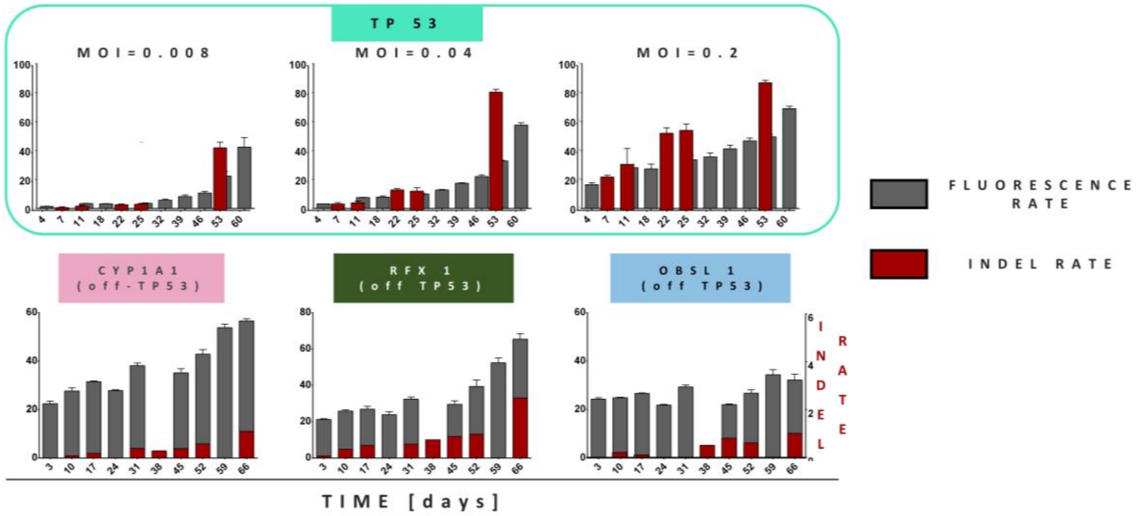

Supplementary Figure S4. Increase in fluorescence rate of the cells transduced with all-in-one lentiviral particles encoding different spacer sequences (TP53, CYP1A1(off TP53), RFX(off TP53) and OBSL(off TP53)) is correlated with indel rate measured by ddPCR. Fluorescence rate is shown in grey and indel rate is shown in red. Only the results OBSL(off TP53) were shown on two different Y axe scales.

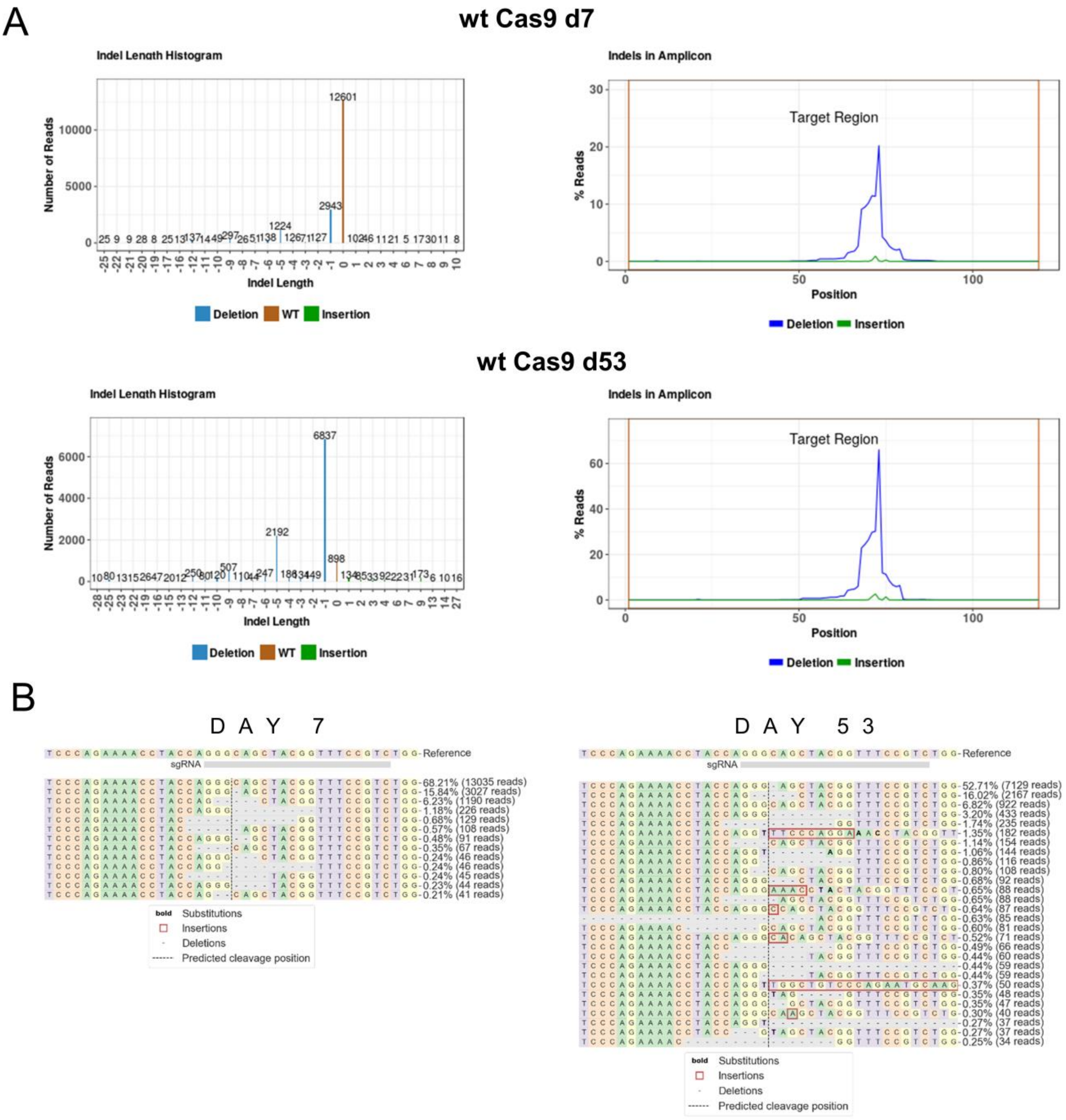

Supplementary Figure S5. NHEJ-mediated Indels within TP53 exon 4 leads to gain of the growth advantage by NUFF cells. **A)** Graphs show Indel length histograms and frequency of the Indels on day 7 and 53 after initial treatment with wt Cas9 and TP53 gRNA. **B)** Graphs obtained with CRISPResso2 shows quality and quantity of Indels found in more than 0.2% of reads day 7 and 53 after initial treatment with wt Cas9 and TP53 gRNA

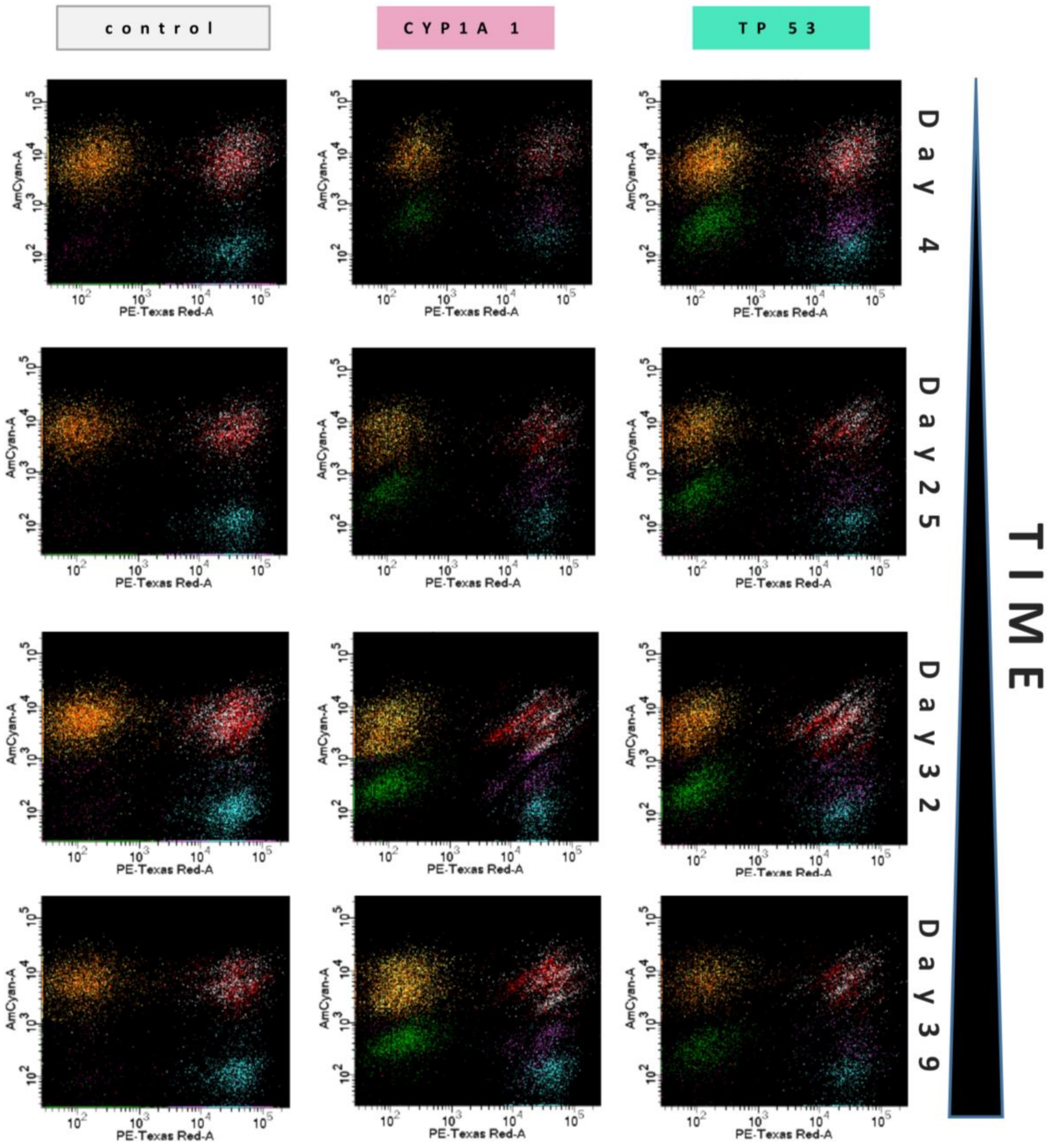

Supplementary Figure S6. Growth advantage gained by the NUFF cells is expressed in clonal growth advantage. Flow cytometry analysis of RGB-marked NUFF cells on week 1, 4, 5, and 6 after initial treatment with wt Cas9 combined with TP53 or A1-1 gRNA. Dominating clones appear on the week 4 in the samples transduced with A1-1 and TP53 gRNAs, however not in the control sample. Clonal domination becomes more pronounced in week 6.

A

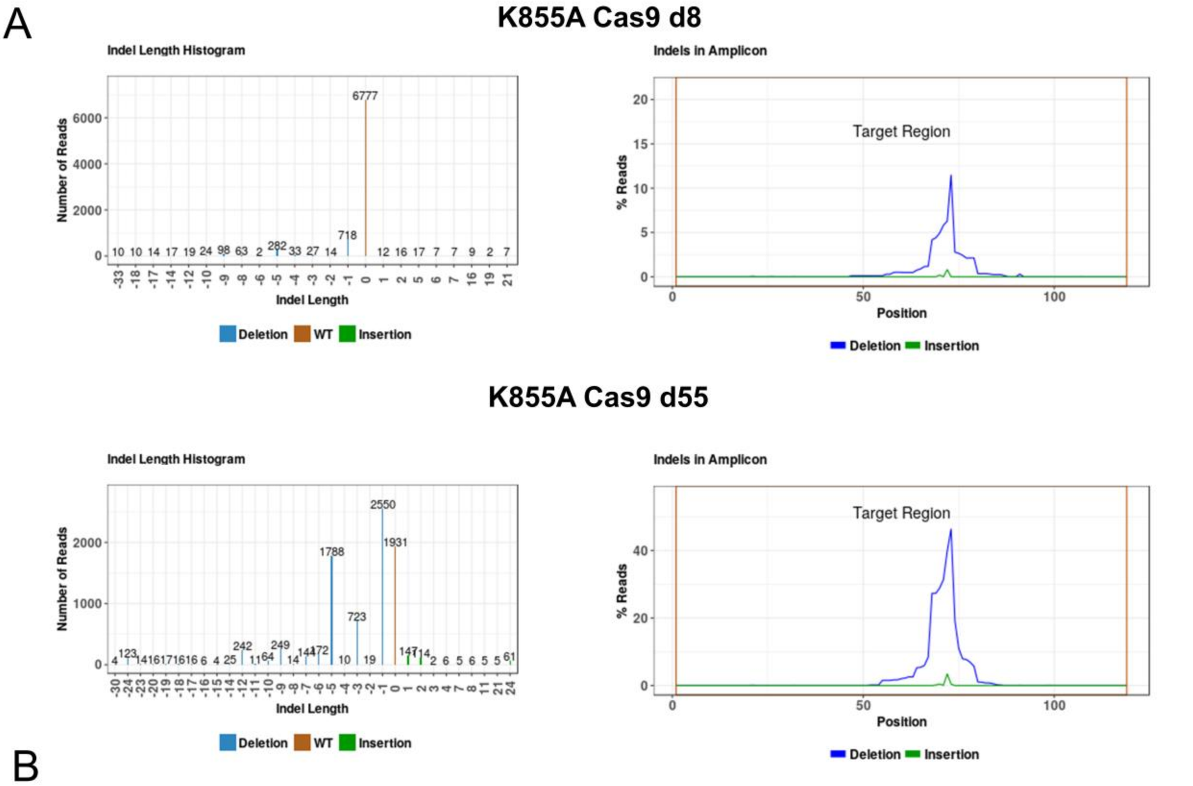

B

	Indel length	Frequency at day 8	Frequency at day 55	Change in %
...GGCCCCTGTCATCTTCTGTCCCTTCCCAGAAAACCTACCAGGGCAGCTACGGTTTCCGTCTGGGCTT...	WT	81.87	21.98	-59.89
...GGCCCCTGTCATCTTCTGTCCCTTCCCAGAAAACCTACCAGGG-AGCTACGGTTTCCGTCTGGGCTT...	-1	8.57	28.18	+19.61
...GGCCCCTGTCATCTTCTGTCCCTTCCCAGAAAACCTACC-----AGCTACGGTTTCCGTCTGGGCTT...	-5	3.01	19.65	+16.64
...GGCCCCTGTCATCTTCTGTCCCTTCCCAGAAAACCTACCAGGG---------TTTCCGTCTGGGCTT...	-9	0.95	2.54	+1.59
...GGCCCCTGTCATCTTCTGTCCCTTCCCAGAAAACCTACCAGGG---CTACGGTTTCCGTCTGGGCTT...	-3	0.32	8.32	+8
...GGCCCCTGTCATCTTCTGTCCCTTCCCAGAAAAC------------CTACGGTTTCCGTCTGGGCTT...	-12	0.23	2.71	+2.41

C

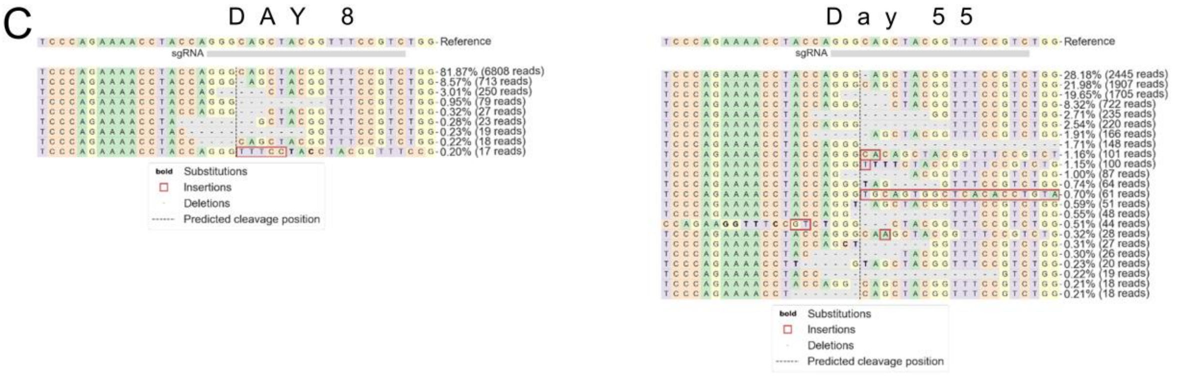

Supplementary Figure S7. Next-generation Cas9 variant's cleavage leads to NHEJ-mediated Indels formation within TP53 exon 4 responsible for the gain of the growth advantage by the NUFF cells. **A)** Graphs show Indel length histograms and frequency of the Indels on day 7 and 53 after initial treatment with Cas9 K855A and TP53 gRNA**. B)** The 5 most frequently found clones on day 8 and 55 are marked with dark gray. Observed change in frequency between day 7 and 53 was marked in blue if positive and brown if negative. **C)** Graphs obtained with CRISPResso2 shows quality and quantity of Indels found in *TP53* in more than 0.2% of NGS reads at day 8 and 55 after initial treatment with Cas9 K855A and TP53 gRNA.
